# Gene expression profiling of early intervertebral disc degeneration reveals a down-regulation of canonical Wnt signaling and caveolin-1 expression: implications for development of regenerative strategies

**DOI:** 10.1186/ar4157

**Published:** 2013-01-29

**Authors:** Lucas A Smolders, Björn P Meij, David Onis, Frank M Riemers, Niklas Bergknut, Richard Wubbolts, Guy CM Grinwis, Martin Houweling, Marian JA Groot Koerkamp, Dik van Leenen, Frank CP Holstege, Herman AW Hazewinkel, Laura B Creemers, Louis C Penning, Marianna A Tryfonidou

**Affiliations:** 1 Department of Clinical Sciences of Companion Animals, Faculty of Veterinary Medicine, Utrecht University, Yalelaan 108, 3508 TD, Utrecht, The Netherlands; 2 Department of Clinical Sciences, Division of Small Animals, Faculty of Veterinary Medicine and Animal Sciences, Swedish University of Agricultural Sciences, Ulls väg 14 C, SE-75651 Uppsala, Sweden; 3 Department of Biochemistry and Cell Biology, Faculty of Veterinary Medicine, Utrecht University, Yalelaan 1, 3584 CL, Utrecht, the Netherlands; 4 Department of Pathobiology, Pathology Division, Faculty of Veterinary Medicine, Utrecht University, Yalelaan 108, 3508 TD, Utrecht, The Netherlands; 5 Molecular Cancer Research, University Medical Center Utrecht, Utrecht, Heidelberglaan 100, 3584 CX, Utrecht, The Netherlands; 6 Department of Orthopedics, University Medical Center Utrecht, Heidelberglaan 100, 3584 CX, Utrecht, The Netherlands

## Abstract

**Introduction:**

Early degeneration of the intervertebral disc (IVD) involves a change in cellular differentiation from notochordal cells (NCs) in the nucleus pulposus (NP) to chondrocyte-like cells (CLCs). The purpose of this study was to investigate the gene expression profiles involved in this process using NP tissue from non-chondrodystrophic and chondrodystrophic dogs, a species with naturally occurring IVD degeneration.

**Methods:**

Dual channel DNA microarrays were used to compare 1) healthy NP tissue containing only NCs (NC-rich), 2) NP tissue with a mixed population of NCs and CLCs (Mixed), and 3) NP tissue containing solely CLCs (CLC-rich) in both non-chondrodystrophic and chondrodystrophic dogs. Based on previous reports and the findings of the microarray analyses, canonical Wnt signaling was further evaluated using qPCR of relevant Wnt target genes. We hypothesized that caveolin-1, a regulator of Wnt signaling that showed significant changes in gene expression in the microarray analyses, played a significant role in early IVD degeneration. Caveolin-1 expression was investigated in IVD tissue sections and in cultured NCs. To investigate the significance of Caveolin-1 in IVD health and degeneration, the NP of 3-month-old Caveolin-1 knock-out mice was histopathologically evaluated and compared with the NP of wild-type mice of the same age.

**Results:**

Early IVD degeneration involved significant changes in numerous pathways, including Wnt/β-catenin signaling. With regard to Wnt/β-catenin signaling, *axin2 *gene expression was significantly higher in chondrodystrophic dogs compared with non-chondrodystrophic dogs. IVD degeneration involved significant down-regulation of *axin2 *gene expression. IVD degeneration involved significant down-regulation in Caveolin-1 gene and protein expression. NCs showed abundant caveolin-1 expression *in vivo *and *in vitro*, whereas CLCs did not. The NP of wild-type mice was rich in viable NCs, whereas the NP of Caveolin-1 knock-out mice contained chondroid-like matrix with mainly apoptotic, small, rounded cells.

**Conclusions:**

Early IVD degeneration involves down-regulation of canonical Wnt signaling and Caveolin-1 expression, which appears to be essential to the physiology and preservation of NCs. Therefore, Caveolin-1 may be regarded an exciting target for developing strategies for IVD regeneration.

## Introduction

Degeneration of the intervertebral disc (IVD) is a major cause of low back pain, a major health problem in the Western world [1]. Low back pain resulting from IVD degeneration may be treated medically in combination with physiotherapy. Surgical therapies include decompression with partial discectomy (removal of the diseased IVD tissue), spinal fusion of the affected segment, or partial or total artificial IVD replacement [2,3]. Although these surgical therapies are generally successful, they are suboptimal since they are not curative and are associated with various complications: decompression/partial discectomy results in spinal instability, spinal fusion can result in adjacent segment degeneration, and IVD replacements/prostheses are associated with failure of the surgical implants [4-9]. Therefore, within the field of regenerative medicine the focus has been on strategies concentrating on biological repair of the degenerating disc using adult stem or progenitor cells, growth factors, and/or gene therapy [10]. However, the biomolecular events involved in IVD degeneration remain largely unexplored [11-13].

Like humans, dogs suffer from spontaneous IVD degeneration that involves similar macroscopic (for example, dehydration of the NP (nucleus pulposus), decrease in disc height), histopathological (for example, chondrocyte proliferation, disorganization of the annulus fibrosus), and biochemical changes (for example, decrease in proteoglycan content, increase in matrix metalloproteinase (MMP) activity) [14]. In humans and dogs, the juvenile, healthy NP of the IVD consists of a dense population of notochordal cells (NCs) embedded in a modest amount of extracellular matrix [15,16]. The NC has restorative capacity upon other cells, such as chondrocyte-like cells (CLCs) and mesenchymal stem cells, with significant regenerative potential, and thus is an interesting focus for regenerative strategies [13,17-21]. In humans and dogs, aging and early degeneration of the IVD involves chondroid metaplasia of the NP, which is characterized by the replacement of NCs by CLCs [11,16,22]. With regard to this cellular phenomenon, the dog is a unique species, because two subspecies can be distinguished, namely, chondrodystrophic and non-chondrodystrophic dog breeds [22,23]. Chondrodystrophic breeds are characterized by a disturbed endochondral ossification, resulting in disproportionally short limbs relative to the length of the spine. In these breeds, replacement of NCs by CLCs occurs before 1 year of age, with a concurrent onset of IVD degeneration at all spinal levels [22]. In contrast, non-chondrodystrophic breeds have normal growth of the long bones, and in these dogs the NC remains the predominant cell type of the NP until middle or old age. In non-chondrodystrophic dogs, IVD degeneration generally occurs at older ages compared with chondrodystrophic dogs (> 4 to 5 years of age), and mainly at selected locations (caudal cervical and lumbosacral spine), probably due to a high workload at these spinal levels [22,24-29].

Therefore, these two dog types reflect a naturally occurring animal model for IVD degeneration, representing differential maintenance of the NC with differential causative factors [14,30]. Hence, the dog can be considered a unique model for studying the (patho)physiology of the NC and associated early IVD degeneration.

Although the process of early IVD degeneration has been described histopathologically [14,16,22], biomolecular signaling pathways involved in the transition from the NP rich in NCs to the NP rich in CLCs (that is, early IVD degeneration) require further investigation. The aim of this study was to investigate the biomolecular signaling profiles associated with NC maintenance and replacement of NCs by CLCs in non-chondrodystrophic and chondrodystrophic dogs, to identify possible targets for IVD regeneration. In the present study, apart from expected biomolecular signaling pathways involved in early IVD degeneration, including Wnt/β-catenin signaling, new pathways were identified. In particular, Caveolin-1, a regulator of Wnt/β-catenin signaling, was found to be a crucial factor in the maintenance of NC health and physiology, and in the initiation of IVD degeneration, being significantly different between the two canine subspecies. These results indicate that Caveolin-1 is an exciting target for further studies.

## Materials and methods

### Ethics statement

All materials used in this study were collected from animals euthanized in other, unrelated experiments approved by the Ethics Committee on Animal Experimentation (DEC) of Utrecht University. Canine IVD tissue was collected from dogs euthanized in studies investigating osteoarthritis (Experiment numbers DEC 2007.III.08.110 and DEC 2009.III.05.037; euthanasia performed by way of an intravenous overdose of pentobarbital) [31-33] and cardiovascular disease (Experiment number DEC 2007.II.01.029; euthanasia performed under general anesthesia by way of fibrillation and subsequent excision of the heart) [[34], other, unpublished data].

Murine IVD tissue was collected from mice euthanized for studies investigating the role of Caveolin-1 in liver regeneration (Experiment number DEC 2008.III.01.001; euthanasia performed by way of exsanguination under isoflurane anesthesia; work not yet published). In these unrelated experiments, the animals were sacrificed for the collection of tissue other than IVD. All experimental procedures were performed strictly according the guidelines set by the Ethics Committee of Utrecht University. The Ethics Committee of Utrecht University approved post-mortem harvesting of the IVD tissue employed in the present study.

### Sample collection

Cervical (vertebrae C2 to T1) and thoracolumbar (vertebrae T10 to S1) spines were collected from five mongrel dogs (non-chondrodystrophic group, age range 13 to 60 months, body weight range 26.6 to 32.1 kg) and six Beagle dogs (chondrodystrophic group, age range 25 to 36 months, weight range, 13.6 to 16.0 kg). Spines were resected *en bloc *and cut into single functional spinal units (FSUs; endplate-IVD-endplate). Each FSU was cut in the sagittal plane into two equal parts. The NP was collected from one part, snap frozen in liquid nitrogen, and stored at -70°C. The other part was fixed in 4% neutral buffered formaldehyde, decalcified in 10% ethylenediaminetetraacetic acid (EDTA) for 3 months at room temperature, and embedded in paraffin.

### Histopathological grading of IVD samples

**Figure 1:**
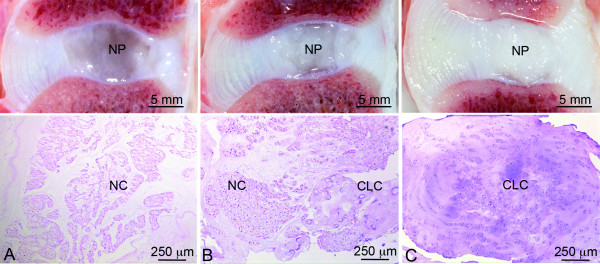
**Typical macroscopic pictures and corresponding H&E sections of the applied classification**. The notochordal cell (NC)-rich nucleus pulposus (NP) (**A**) contains NCs with a viable morphology organized in clusters; the mixed group (**B**) contains both NCs and chondrocyte-like cells (CLCs); and the CLC-rich group (**C**) contains solely CLCs embedded in a dense matrix.

To optimally investigate mRNA expression patterns associated with NC maintenance and replacement of NCs by CLCs, histopathological grading was performed as described previously [35]. Mid-sagittal sections (4 μm) were mounted on Microscope KP+ slides (Klinipath B.V, Duiven, the Netherlands) and stained with H&E. Composite raw images of each IVD were made using a Colorview IIIU digital camera (Olympus, Zeist, the Netherlands) mounted to a BX-40 microscope (Olympus, Zeist, the Netherlands). The images were scaled and the following parameters were measured for the NP: 1) proportion (%) of NP surface area and pericellular matrix consisting of NCs, which were identified based on morphologic characteristics (cell size, intracytoplasmic vesicles, typical NC clusters) [16,36], and 2) proportion of NP surface area consisting of CLCs and fibrocartilaginous matrix. By combining these parameters for each IVD sample, samples were assigned by a board-certified veterinary pathologist to one of three groups, namely, 1) a notochordal cell-rich (NC-rich) group (> 90% of NP surface area consisting of NCs), 2) a mixed group (cell population consisting of both NCs and CLCs, with 10% to 90% of NP surface area consisting of NCs), and a CLC-rich group (> 90% of NP surface area consisting of CLCs and corresponding matrix) (Figure 1).

To further assess the cellular phenomenon involving the transition from NC-rich to CLC-rich NP, and thus the applied histopathological grading, the gene expression of the notochordal markers *Brachyury *[12,37] and *Cytokeratin 8 *[38,39] was investigated in all groups (qPCR analysis, see below).

### DNA microarray analysis

Four NPs from the three histopathological groups for each breed type were randomly selected and used to isolate total RNA, using the RNeasy Fibrous Tissue Mini Kit (Qiagen, Venlo, the Netherlands) according to the manufacturer's instructions. After on-column DNase-I treatment (Qiagen RNAse-free DNase kit, Venlo, the Netherlands), RNA was quantified spectrophotometrically using Nanodrop ND-1000 (Isogen Life Science, De Meern, the Netherlands) and RNA integrity was determined using a Bioanalyzer 2100 (Agilent Technologies, Amstelveen, the Netherlands). RNA integrity numbers of the samples varied from 4.5 to 7.4 (mean ± SD 6.2 ± 0.7), indicating that these samples could be used to perform valid gene expression analysis [40].

A two-color DNA microarray with a reference experiment design was performed on 44 k Canine Gene Expression Microarrays V1 (G2519F, Agilent Technologies, Amstelveen, the Netherlands) [41]. Double round RNA amplification and labeling were performed as described before [42] on an automated system (Caliper Life Sciences, 's-Hertogenbosch, the Netherlands) with 10 to 50 ng total RNA input from each sample. The common RNA reference pool consisted of a multitude of canine organs, including liver, spleen, kidney, lung, heart, intestine, and bone. Hybridizations were performed on an HS4800PRO system with QuadChambers (Tecan Benelux B.V.B.A., Giessen, the Netherlands), using 300 to 500 ng labeled cRNA per channel as described previously [43]. From each group two samples were labeled with Cy5 and hybridized against the common reference cRNA (Cy3) on dual channel arrays, and two samples were hybridized in dye swap. Hybridized slides were scanned on an Agilent scanner (G2565BA) at 100% laser power, 100% photomultiplier tube (PMT). After automated data extraction using Imagene 8.0 (BioDiscovery, El Segundo, CA, United States of America), print-tip loess normalization was performed [44] on mean spot intensities. Dye bias was corrected based on a within-set estimate [45]. Patterns of gene expression were compared between the three stages of IVD degeneration (NC-rich, mixed, CLC-rich NP) within each breed type and between the two breed types. Data were analyzed using microarray analysis of variance (MAANOVA) [46]. In a fixed-effect analysis, sample, array, and dye effects were modeled. *P*-values were determined with a permutation F2-test, in which residuals were shuffled 5,000 times globally. Genes with a *P*-value < 0.05 after Benjamini-Hochberg determination of false discovery rate (FDR) were considered significantly changed; a change cutoff value of 1.3-fold was applied.

Differentially expressed genes were converted to their human homologues, and the following array comparisons were included in functional pathway analysis using the GeneGo MetaCore platform [47]: 1) NC-rich group vs. mixed group, mixed group vs. CLC-rich group, and NC-rich group vs. CLC-rich group in both chondrodystrophic and non-chondrodystrophic dogs; 2) non-chondrodystrophic vs. chondrodystrophic breeds: NC-rich group, mixed group, and CLC-rich group. Pathways showing significant changes in gene expression were selected and analyzed further as described below.

### Quantitative PCR (qPCR)

Six NPs from the three histopathological groups for each breed type were analyzed (cDNA samples used for microarray and additional, similarly processed samples). Unlabeled microarray cDNA from the first round of amplification was used. For the additional samples, cRNA was synthesized by extracting RNA as described for the microarray, followed by one round of amplification using *in vitro *transcription [48], assuring similar pre-PCR treatment of both cRNA sources.

For all samples, cDNA was synthesized using the iScript™ cDNA Synthesis Kit (Biorad, Veenendaal, the Netherlands). qPCR was performed using a MyIQ thermal cycler, IQ SYBRGreen SuperMix (BioRad, Veenendaal, the Netherlands) and dog-specific primers (Eurogentec, Maastricht, the Netherlands) (see Additional file 1). Each sample was treated as an individual sample, and samples from different histopathological grades and subspecies were analyzed separately (in tehnical duplicates). Primers were designed for the notochordal marker genes *Brachyury *and *Cytokeratin 8*, and based on the microarray analyses primers were designed for the following Wnt target genes: *Wnt3a*, *Wnt7b, Wnt inhibitory factor 1 (Wif1), Frizzled 1 (Fzd1), Low density lipoprotein receptor-related protein 5 (Lrp5), Dickkopf homolog 3 (Dkk3), Integrin-linked kinase (Ilk), Caveolin-1 (Cav1) *and *Axin2 *[49-57]. Conditions for the qPCR experiments were carefully validated as described previously [35]. The amplification efficiency was between 90% and 110%. Relative expression was calculated by the efficiency-corrected delta-delta Ct (ΔΔCt) method [58] using a set of five reference genes (see Additional file 1).

Using the ΔΔCt as a parameter value, linear mixed models [59,60] were designed to statistically analyze the obtained data for each individual target gene. *P*-values were calculated to analyze differences in relative gene expression between groups (chondrodystrophic and non-chondrodystrophic dogs) and degeneration stages (NC-rich, mixed, CLC-rich). The Benjamini-Hochberg correction was used to correct for multiple comparisons [61]. *P *< 0.05 was considered statistically significant. For a complete description of the statistical analyses, see Additional file 2.

### β-Catenin protein expression

To validate differences in Wnt/β-catenin signaling between non-chondrodystrophic and chondrodystrophic dogs, immunohistochemistry of β-catenin was performed as described before [35], and analyzed focussing on differences between non-chondrodystrophic and chondrodystrophic dogs. Integrated density for β-catenin staining of the NP was corrected for the surface area of the NP, positively stained for β-catenin, in order to correct for the difference in disc size between the two breed groups. Furthermore, total protein was extracted from lumbar CLC-rich NPs from non-chondrodystrophic and chondrodystrophic dogs using radioimmunoprecipitation assay (RIPA) buffer; protein concentration was determined with a Lowry assay. Aliquots of protein were subjected to 10% SDS-PAGE (15 μg/lane). Protein (5 μg) from a human insulinoma CM cell line [62] served as positive control. The proteins were electroblotted onto a Hybond-C nitrocellulose membrane (Amersham Biosciences, RPN203C, Bath, United Kingdom). After blocking with 4% non-fat dry milk in Tris-buffered saline containing 0.1% Tween20 (TBST0.1), the membrane was incubated overnight at 4°C with β-catenin antibody (Ab6302, Abcam, Cambridge, United Kingdom, 1:500 in 4% BSA in TBST0.1). After three 5-minute washes in TBST0.1, the membrane was incubated for 90 minutes with anti-rabbit horseradish peroxidase (HRP)-conjugated secondary antibody (R&D, Minneapolis, MN, United States of America; HAF008, 1:20.000 in TBST0.1). Protein expression was detected using an enhanced chemiluminescence substrate (ECL Advance, Amersham RPN2135, Bath, United Kingdom) in a ChemiDoc XRS System (Bio-Rad Laboratories, Veenendaal, the Netherlands). Control experiments were included by omitting the primary antibodies. After completing the western blot for β-catenin, the membranes were washed in TBST0.1 and re-used to determine α-tubulin protein expression. For this purpose, all steps as described above were performed except for the antibody incubations: the membrane was incubated with primary antibody for α-tubulin (Sigma T6199, 1:750, Zwijndrecht, the Netherlands) for 2 hours at room temperature, followed by secondary anti-mouse HRP-conjugated antibody (R&D HAF007,1:20000, Minneapolis, MN, United States of America). All experiments were performed in triplicate.

### Caveolin-1 immunohistochemistry

Paraffin-embedded IVD (as described above) sections from five non-chondrodystrophic and five chondrodystrophic dogs for each group (NC-rich, mixed, CLC-rich; *n *= 10 per group) were subjected to antigen retrieval in 10 mM citrate buffer (pH 6.0), followed by blocking of endogenous peroxidase activity [35]. Nonspecific background staining was minimized by pre-incubation with blocking buffer containing 10% normal goat serum (Sigma-Aldrich, Zwijndrecht, the Netherlands)/0.1% Tween-20 (Tween-20, Boom BV, Meppel, the Netherlands) in PBS for 30 minutes, and an overnight incubation at 4^°^C with the primary antibody monoclonal mouse anti-Caveolin-1 (Transduction Laboratories, Breda, the Netherlands mAb2297, 2.5 μg/ml, 1:100 in PBS with 0.1% Tween-20). After sections were washed in PBS buffer/0.025% Triton X, Caveolin-1 was visualized with the goat anti-mouse Envision System and the liquid diaminobenzidine (DAB) chromogen system (Dako, Heverlee, Belgium) and counterstained with hematoxylin (Hematoxylin QS, Vector Laboratories Inc., Peterborough, United Kingdom). In negative control sections, the primary antibody was omitted or replaced with its respective serum. All sections were stained in the same session.

Detailed overview images of each stained slide were made using a Colorview IIIU digital camera (Olympus, Zeist, the Netherlands) mounted to a BX-40 microscope (Olympus, Zeist, the Netherlands). The total NP surface of each sample was measured by defining the perimeter of the NP, excluding the transition zone. A custom-made color range selection optimized for Caveolin-1 specific staining was used to calculate the proportion of the NP surface area that stained for Caveolin-1, and the mean gray value (staining intensity) for Caveolin-1 staining in each sample, as described previously [35].

For each parameter, linear mixed models were used to calculate *P*-values to analyze differences between groups (chondrodystrophic and non-chondrodystrophic) and degeneration stages (NC-rich, mixed, CLC-rich) (for a complete description of the statistical analyses: Additional file 2). The Benjamini-Hochberg correction was used to correct for multiple comparisons [61]. *P *< 0.05 was considered statistically significant.

To investigate the relationship between Caveolin-1 and canonical Wnt signaling in the NP, their colocalization was investigated by way of simultaneous immunofluorescence analysis of both proteins. Paraffin-embedded IVD slides were used for immunofluorescent labeling of Caveolin-1 as described above, except that the slides were incubated overnight with a combination of primary antibodies monoclonal mouse anti-Caveolin-1 (Transduction Laboratories, Breda, the Netherlands, mAb2297, 2.5 μg/ml, 1:100 in PBS with 0.1% Tween-20) and polyclonal rabbit anti-β-catenin (Abcam, Cambridge, United Kingdom, ab6302, 1:50 in PBS with 0.1% Tween-20). The secondary antibodies used were 1:100 goat anti-mouse antibody conjugated to Alexa488 (2.5 μg/ml; Invitrogen, Breda, the Netherlands) and 1:100 goat anti-rabbit antibody conjugated to Alexa568 (2.5 μg/ml; Invitrogen, Breda, the Netherlands). Topro-3 iodide (2 μg/ml; Invitrogen, Breda, the Netherlands, T3605) was used to stain the nucleus.

To outline the proximity of the fluorescently-marked Caveolin-1 and β-catenin proteins, profile intensity plots were generated in LAS-AF imaging software (Leica microsystems, Wetzlar, Germany). Straight lines were drawn across representative cell bodies and intensity profiles were extracted from the channels visualizing and measuring the fluorescence of Caveolin-1, β-catenin, and Topro-3. The data were subsequently exported to Microsoft Excel (Microsoft Corporation, Amsterdam, the Netherlands) and plotted.

### Caveolin-1 expression in NCs in vitro

On the basis of the previous analyses, Caveolin-1 was investigated in NCs *in vitro*. NCs were isolated from the NPs of cervical (C2 to T1) and lumbar (L1 to S1) IVDs from six, young-adult, mongrel dogs (non-chondrodystrophic, aged 16 to 18 months, and weighing 16 to 24 kg). The NCs were cultured in their original cluster-like conformation as described previously [35] on coverslips in 6-well plates (Falcon Multiwell Primaria, Becton Dickinson, Breda, the Netherlands) in penicillin/streptomycin (P/S)-FCS, 10%)-DMEM-F12 under normoxic conditions (5% CO_2_) at 37°C for 10 days.

For RNA isolation, non-adherent cells at days 0 and 2 (NCs first adhered on day 4) were collected by centrifuging the medium at 1500 RPM at 4°C for 1 minute; on days 4, 6, 8, and 10, the medium was removed, the wells were washed with RNase-free Hank's solution, and the adherent cells were lysed and used for analysis. For all time points, total cellular RNA was isolated using the RNeasy Mini Kit (Qiagen, Venlo, the Netherlands) according to the manufacturer's instructions. The relative gene expression of *Caveolin-1 *was analyzed as described above.

Non-adhered cells on days 0 and 2 were collected from the culture medium and mounted on positively charged slides (Klinipath, Duiven, the Netherlands) using a Shandon Cytospin 4 (Therma Scientific, Landsmeer, the Netherlands). Cells were fixed on days 0, 2, 4, 6, 8, and 10, and were used for immunofluorescent labeling of Caveolin-1 as described above, except that the secondary antibody used was 1:100 donkey anti-mouse antibody conjugated to Alexa488, (2.5 μg/ml; Invitrogen, Breda, the Netherlands). Topro-3 iodide (2 μg/ml; Invitrogen, Breda, the Netherlands, T3605) was used to stain the nucleus. The cells were mounted with Fluorsave (Calbiochem, Darmstadt, Germany). Images at 5 random locations in each sample were acquired by a sequential recording procedure on a multiphoton imaging station (MP2100, Zeiss, Herfordshire, United Kingdom). Immunofluorescent images of the cells were analyzed with CellProfiler 2.0 software package (Massachusetts Institute of Technology, Massachusetts, United States of America), with cell nuclei detected on the Topro-3 images (nuclear staining) and the Caveolin-1 expression signal on the Caveolin-1 images. For each image, the total number of cells and the total Caveolin-1 signal were measured, and the estimated mean intensity of Caveolin-1 protein staining per cell was calculated by dividing the total Caveolin-1 signal, after subtraction of background, by the total number of cells.

For both ΔCT for *Caveolin-1 *gene expression, and Caveolin-1 protein expression per cell, linear mixed models were designed to calculate *P*-values to analyze differences in the Caveolin-1 gene and protein expression between time points in culture. The Benjamini-Hochberg correction was used to correct for multiple comparisons [61]. *P *< 0.05 was considered statistically significant (see Additional file 2 for a complete description of the statistical analyses).

### NP in Caveolin-1 knock-out (KO) mice

To further assess the role of Caveolin-1 in NC preservation and early IVD degeneration, spines were collected from 3-month-old Caveolin-1 KO-mice (*Cav^tm1Mls ^*, JAX^®^, the Jackson Laboratory, Maine, United States of America) and wild-type mice (strain B6129SF2, JAX^®^). Spines were fixed in 4% neutral buffered formalin, decalcified (7 days in EDTA at 4°C), and embedded in paraffin. Mid-sagittal sections (4 μm) were mounted on Microscope KP+ slides (Klinipath B.V., Duiven, the Netherlands) and stained with H&E and alcian blue/picosirius red, the latter highlighting proteoglycan (blue) and collagen content (red) [63]. Multiple sections of the NP of multiple cervical and lumbar IVDs (*n *= 4) from each mouse were histopathologically evaluated.

## Results

### Microarray: changes in gene expression

In NPs from non-chondrodystrophic dogs, the NC-rich, mixed, and CLC-rich groups consisted of 100.0% NCs, 45.4% to 87.0% NCs, and 100.0% CLCs, respectively; in NPs from chondrodystrophic dogs, these proportions were 93.9% to 100.0% NCs, 39.7% to 89.9% NCs, and 100.0% CLCs, respectively. Therefore, the applied histopathological classification resulted in the NC-rich, mixed, and CLC-rich groups being comparable between chondrodystrophic and non-chondrodystrophic dogs, allowing reliable investigation of early IVD degeneration within both types of breed, and comparison between the two types of breed.

**Figure 2:**
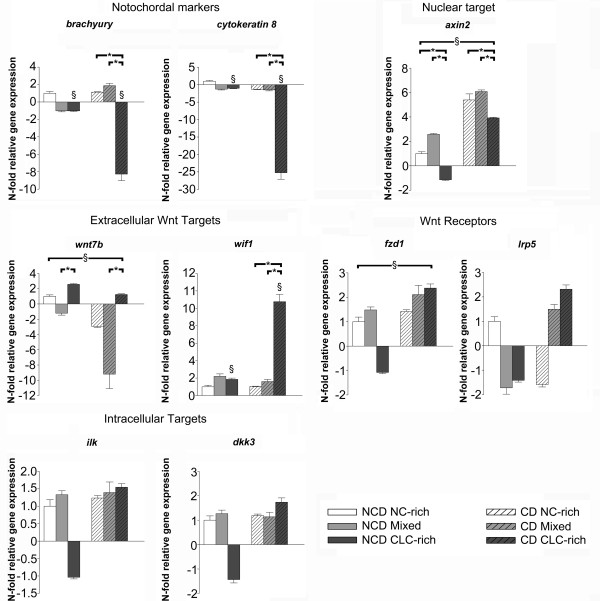
**Relative gene expression of relevant target genes**. Relative gene expression of *Brachyury*, *Cytokeratin 8, Axin2*, *Frizzled 1 *(*Fzd1*), *Low density lipoprotein receptor-related protein 5 *(*Lrp5*), *Wnt7b*, *Wnt inhibitory factor 1 (Wif1)*, *Integrin linked kinase *(*Ilk*), and *Dickkopf homolog 3 *(*Dkk3*) in the notochordal cell-rich (NC-rich), mixed, and chondrocyte-like cell rich (CLC-rich) NP from non-chondrodystrophic (NCD) and chondrodystrophic (CD) dogs (NCD, NC-rich NP was used as reference, set at 1). *Significant difference between NC-rich, mixed, and CLC-rich NP; ^§^significant difference between NCD and CD dogs.

qPCR analysis revealed no significant differences in the expression of the notochordal markers *Brachyury *and *Cytokeratin 8 *in the different histopathological stages in non-chondrodystrophic dogs, indicating that the expression of NC marker genes was preserved in all histopathological stages despite significant changes in IVD morphology (Figure 2 and Additional file 3). However, in chondrodystrophic dogs, *Brachyury *and *Cytokeratin 8 *gene expression was significantly downregulated in the CLC-rich group compared with the NC-rich and mixed groups; *Brachyury *and *Cytokeratin 8 *gene expression was significantly lower in CLC-rich NP from chondrodystrophic dogs than in CLC-rich NP from non-chondrodystrophic dogs. These results suggest that in chondrodystrophic dogs the transition from NC-rich to CLC-rich NP involves a significant downregulation in NC marker gene expression. These results were sustained by the microarray results, showing decreased gene expression in the CLC-rich group compared with the NC-rich group of notochordal markers *Cytokeratin 8 *and *19 *[38,64,65].

Numerous up- and downregulated genes were found in the microarrays (Table 1 and Additional file 4; the microarray data discussed in this manuscript have been deposited in NCBI's Gene Expression Omnibus (GEO) [66] [GEO: GSE35717] [67]. Metacore pathway map analysis showed that several signaling pathways were up- or downregulated in the transition from NC-rich to CLC-rich NP from chondrodystrophic dogs, such as extracellular matrix remodeling, plasmin signaling, plasminogen activator-urokinase (PLAU)-signaling, bone morphogenetic protein signaling, and Wnt signaling/cytoskeletal remodeling (see Additional file 5).

**Table 1:** Top 25 up- and downregulated genes for the microarray comparison of notochordal cell (NC)-rich nucleus pulposus (NP) (reference) vs. chondrocyte-like cell-(CLC)-rich NP in chondrodystrophic dogs

Chondrodystrophic dogs: NC-rich NP vs. CLC-rich NP			
**Total upregulated genes: 1.178**	**Total downregulated genes: 1,741**		

**Description**	**GO term: biological Process**	**N-fold change**	***P*-value**

Carboxypeptidase E	Protein modification process	8.65	8.65E-05
Transferrin	Transferrin transport	8.58	1.56E-02
Ceruloplasmin (ferroxidase)	Cellular iron ion homeostasis	7.34	2.02E-03
Frizzled-related protein	Negative regulation of canonical Wnt receptor signaling pathway	7.26	4.24E-03
Decorin	Peptide cross-linking via chondroitin 4-sulfate glycosaminoglycan	7.11	2.00E-02
Cartilage oligomeric matrix protein	Anti-apoptosis	7.08	6.69E-05
Serglycin	Negative regulation of bone mineralization	6.97	9.64E-03
Lumican	Collagen fibril organization	6.88	1.39E-02
Not annotated	Not annotated	6.66	1.15E-02
Metallothionein 2A	Cellular response to erythropoietin	6.60	5.33E-03
RAN binding protein 3-like	Intracellular transport	6.54	4.71E-02
Retinol binding protein 4, plasma	Protein complex assembly	6.40	6.79E-04
Cysteine dioxygenase, type I	Response to glucagon stimulus	6.30	1.36E-02
Adenylate cyclase 2	Activation of adenylate cyclase activity by G-protein signaling pathway	5.97	3.94E-04
Tetraspanin 13	Not available	5.88	1.95E-02
Microfibrillar associated protein 5	Not available	5.75	1.17E-04
Proteoglycan 4	Cell proliferation	5.61	3.00E-03
S100 calcium binding protein A12	Inflammatory response	5.59	1.15E-02
Phosphotyrosine interaction domain containing 1	Not available	5.57	8.41E-05
Nephronectin	Cell differentiation	5.54	2.76E-04
Lysozyme	Cell wall macromolecule catabolic process	5.47	2.42E-02
SPARC-like 1 (hevin)	Signal transduction	5.34	2.85E-02
Glycoprotein 25L	Not available	5.27	5.84E-04
Serpin peptidase inhibitor, clade G (C1 inhibitor), member 1	Regulation of proteolysis	5.08	6.92E-04
Sphingomyelin phosphodiesterase, acid-like 3A	Sphingomyelin catabolic process	5.01	1.16E-02
Keratin 18	Golgi to plasma membrane CFTR protein transport	-12.93	1.44E-03
tRNA-yW synthesizing protein 3 homolog	tRNA processing	-7.83	9.45E-04
A kinase (PRKA) anchor protein 12	Signal transduction	-7.69	7.56E-03
Phospholipase C-like 1	Lipid metabolic process	-7.40	9.48E-03
Desmocollin 3	Cell adhesion	-7.05	1.40E-03
Myosin, light chain 9, regulatory	Regulation of muscle contraction	-6.84	1.64E-02
Mitochondrial ribosomal protein S27	Not available	-6.76	4.96E-04
Ectonucleotide pyrophosphatase/phosphodiesterase 2	Regulation of cell migration	-6.41	9.38E-03
Keratin 19	Cell differentiation involved in embryonic placenta development	-6.32	6.00E-03
Plakophilin 2	Carbohydrate metabolic process	-6.13	6.02E-04
Tetraspanin 7	Interspecies interaction between organisms	-6.01	3.00E-03
Keratin 8	Cytoskeleton organization	-5.94	2.60E-02
Nucleosome assembly protein 1-like 1	DNA replication	-5.92	1.40E-03
RAB20, member RAS oncogene family	Small GTPase mediated signal transduction	-5.79	2.91E-03
Caldesmon 1	Positive regulation of protein binding	-5.68	3.60E-03
Potassium voltage-gated channel, delayed-rectifier, subfamily S, member 3	Synaptic transmission	-5.64	4.46E-03
Apelin	Positive regulation of phosphorylation	-5.59	7.66E-03
Sorbin and SH3 domain containing 2	Biological process	-5.42	3.39E-03
Phosphatidylcholine transfer protein	Cholesterol metabolic process	-5.37	1.30E-03
Kv channel interacting protein 1	Synaptic transmission	-5.14	< 1.0E-06
Carbonic anhydrase II	Carbon utilization	-5.07	2.57E-02
Thy-1 cell surface antigen	Cytoskeleton organization	-4.74	1.10E-02
RAB38, member RAS oncogene family	GTP catabolic process	-4.71	5.79E-04
Sema domain, immunoglobulin domain (Ig), short basic domain, secreted, (semaphorin) 3C	Neural tube development	-4.58	1.01E-04
Desmocollin 2	Cell adhesion	-4.48	9.91E-03

This specific comparison was chosen to illustrate the overall trend in gene regulations observed in early IVD degeneration. For the top 50 up- and downregulations of all performed microarray analyses, see Additional file 4. For brevity, only one Gene Ontology (GO)-term is given for each gene (obtained with bioDBnet [97]).

Metacore pathway map analysis could not be performed on the gene regulation results from non-chondrodystrophic dogs, because relatively too few genes were down- or upregulated in this breed group (see Additional file 4).

Wnt/β-catenin signaling was analyzed further because it is involved in both the regeneration and the degeneration of various tissues [68]. The expression of the *Wnt7b *(Wnt ligand), *Wif1 *(inhibits by binding to Wnt ligands), *Ilk *(inhibits glycogen synthase kinase 3-β), and *Lrp5 *(Wnt co-receptor) genes was significantly changed and these Wnt/β-catenin target genes were analyzed further by qPCR, as were additional targets involved in canonical Wnt signaling: *Wnt3a *(Wnt ligand), *Fzd1 *(Wnt receptor), *Dkk3 *(negative regulator of Wnt), and *Axin2 *(Wnt read-out) (see Additional file 3).

### Quantitative PCR of the canonical Wnt signaling pathway and β-catenin protein expression

The relative gene expression of *axin2*, which is a highly reliable read-out for the activity of Wnt/β-catenin signaling [69-71], was significantly lower in the CLC-rich group than in the NC-rich and mixed groups in both non-chondrodystrophic and chondrodystrophic dogs (Figure 2). In chondrodystrophic dogs, this decrease in *Axin2 *gene expression may be related to the gene expression of *Wif1 *(inhibits Wnt ligands), which was significantly upregulated in the CLC-rich group compared with the NC-rich and mixed groups. In non-chondrodystrophic dogs, no significant changes in *Wif1 *gene expression were found. However, gene expression of the Wnt ligand *Wnt7b*, which activates canonical Wnt signaling through interactions with Fzd and LRP5 [72], was significantly higher in the CLC-rich group compared with the mixed group in both non-chondrodystrophic and chondrodystrophic dogs.

Compared with non-chondrodystrophic dogs, *Axin2 *gene expression was significantly higher in chondrodystrophic dogs in all histopathological groups, indicating an overall higher Wnt signaling activity in chondrodystrophic dogs. Accordingly, the integrated density of β-catenin corrected for the NP surface area positively stained, was significantly higher in the CLC-rich NP of chondrodystrophic dogs compared with non-chondrodystrophic dogs (see Figure S1A and B in Additional file 6, and Additional file 7). Due to the abudance of matrix protein in the native CLC-rich NP tissue, western blot analysis of active β-catenin expression was cumbersome and quantification of the data was not reliable. However, the chondrodystrophic CLC-rich NP appeared to have less degraded β-catenin compared to non-chondroystrophic dogs (see Figure S1C in Additional file 6, and Additional file 7). These findings are sustained by investigation of the gene expression of the Wnt receptor *Fzd1*, which was significantly higher in chondrodystrophic dogs than in non-chondrodystrophic dogs for all three histopathological stages. Gene expression of *Wnt7b *was significantly higher in non-chondrodystrophic dogs compared with chondrodystrophic dogs in all histopathological stages.

The relative gene expression of *Lrp5*, *Ilk*, and *Dkk3 *remained unchanged in both non-chondrodystrophic and chondrodystrophic dogs. Gene expression of the *Wnt3a *was undetectable in all groups in both breed types.

### *Caveolin-1 *expression

The microarray analyses showed significant changes in *Caveolin-1*, -*2 *and -*3*. Early IVD degeneration involved significant downregulation of *Caveolin-1 *and -*2*, and significant upregulation of *Caveolin-3 *(see Additional files 3 and 4). Given the role of *Caveolin-1 *in the regulation of canonical Wnt signaling [55,56,73] and the reported upregulation of *Caveolin-1 *in degenerated human IVDs [74], its gene and protein expression were further investigated by way of qPCR and immunohistochemistry.

**Figure 3:**
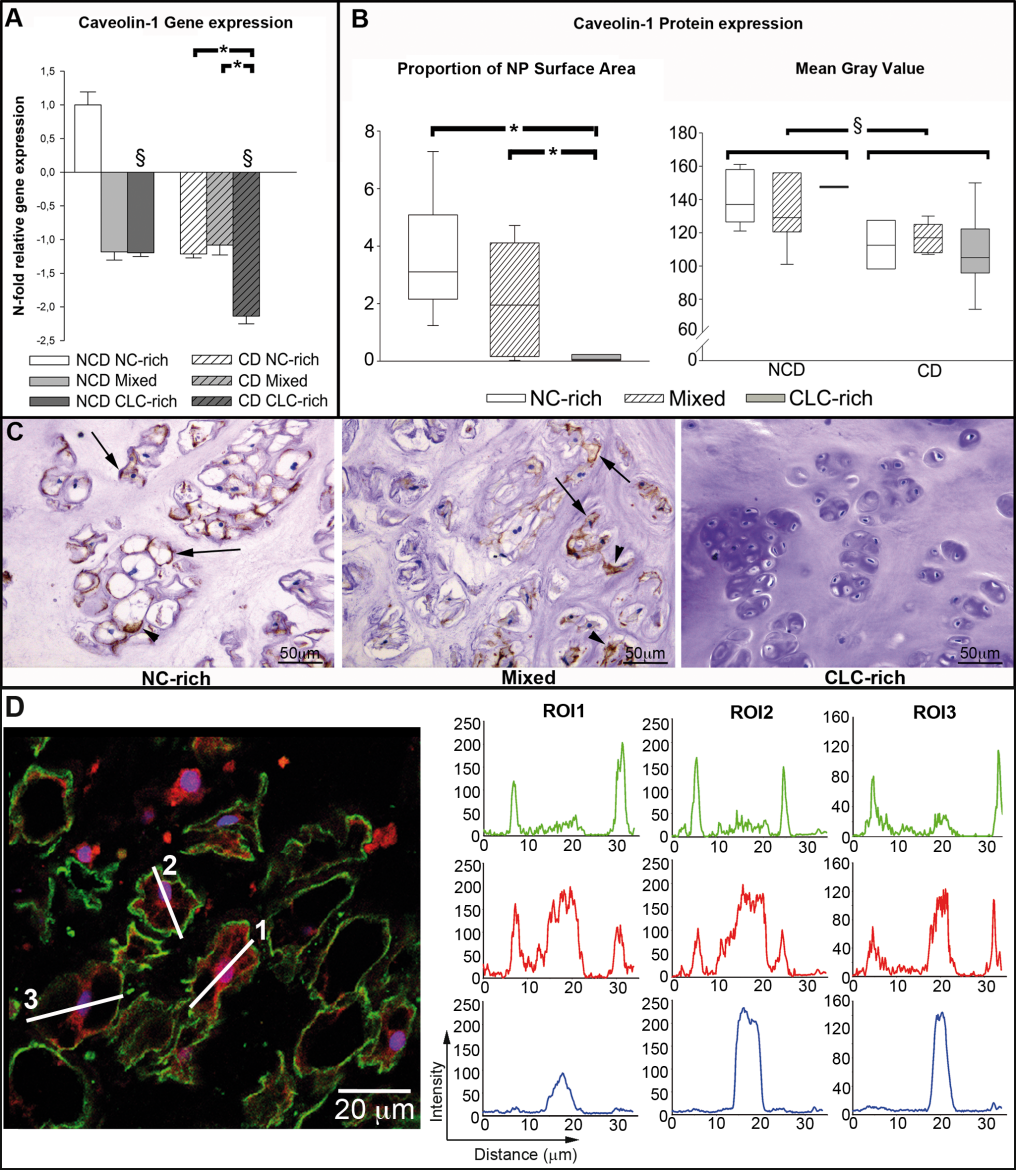
**Caveolin-1 expression in the course of early intervertebral disc degeneration**. (**A**) *Caveolin-1 *gene expression and (**B**) proportion of the NP surface area that stained for Caveolin-1, and mean gray value for Caveolin-1 protein expression in the notochordal cell (NC)-rich, mixed, and chondrocyte-like cell (CLC)-rich nucleus pulposus (NP) from non-chondrodystrophic (NCD) and chondrodystrophic (CD) dogs. *Significant difference between degeneration stages; ^§^significant difference between NCD and CD dogs. The proportion of the NP surface area that stained for caveolin-1 was not divided into NCD and CD dogs because no significant differences were found between breed types. (**C**) Typical examples of NP samples stained for Caveolin-1, showing the NC-rich NP, mixed cell population NP with NCs and CLCs, and the CLC-rich NP. In the NC-rich and mixed groups, membranous (arrow) and cytoplasmic (arrowhead) staining can be observed. Note that Caveolin-1 staining is not observed in CLCs. (**D**) Immunofluorescent staining of the NC-rich NP for the proteins Caveolin-1 (green) and β-catenin (red), and for DNA (blue). Region of interest (ROI) lines drawn across cell bodies were used to generate profile intensity plots (right) for the signal intensity of the Caveolin-1 (green), β-catenin (red), and Topro-3 (blue). The signal intensity peaks for Caveolin-1 correspond with the signal peaks of β-catenin at the cell membrane (located at the same distance of the ROI line), indicating colocalization of these proteins. Also, the central β-catenin signal peaks correspond with the Topro-3 signal peaks, indicating nuclear localization of β-catenin.

In chondrodystrophic dogs, the gene expression of *Caveolin-1 *was significantly downregulated in the CLC-rich compared with the NC-rich and mixed groups (Figure 3); no significant changes were found in non-chondrodystrophic dogs. The gene expression of *Caveolin-1 *in the CLC-rich NP was significantly lower in chondrodystrophic dogs than in non-chondrodystrophic dogs.

Caveolin-1 protein was predominantly located in the cell membranes of NCs, and occasionally in their cytoplasm; Caveolin-1 protein was seldom observed in CLCs. The proportion of the NP surface area that stained for Caveolin-1 was significantly lower in the CLC-rich NP than in the NC-rich and mixed NP in both non-chondrodystrophic and chondrodystrophic dogs, indicating decreased Caveolin-1 protein expression (see Additional file 8). No significant differences were found in the mean gray value (staining intensity) between the NC-rich, mixed, and CLC-rich groups in both non-chondrodystrophic and chondrodystrophic dogs. The intensity of Caveolin-1 staining in all three histopathological groups was significantly higher in non-chondrodystrophic than in chondrodystrophic dogs.

To further assess the relationship between Caveolin-1 and canonical Wnt signaling in the NP, co-immunofluorescence of Caveolin-1 and β-catenin was performed. Profile intensity plots (Figure 3) showed clear signal peaks of β-catenin expression within the cell nucleus of NCs, indicative of active canonical Wnt signaling. Also, clear protein expression of Caveolin-1 and β-catenin was colocalized at the cell membrane of NCs, which is supportive of interaction of these proteins within the NC-rich NP. In CLCs, Caveolin-1 and β-catenin protein expression was rarely observed, which is indicative of less active canonical Wnt signaling and no colocalization and potential protein interaction.

### To verify the possible role of Caveolin-1 in NCs, its expression and intracellular distribution in primary NCs was investigated

**Figure 4:**
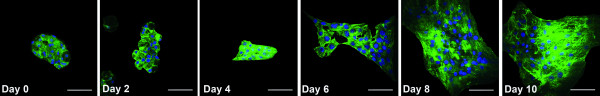
**Immunofluorescence of Caveolin-1 in primary notochordal cells in monolayer culture**. Immunofluorescence images of the notochordal cell clusters on days 0, 2, 4, 6, 8, and 10 in culture. Scale bar: 50 μm. Nuclear staining (Topro-3) and Caveolin-1 staining are depicted in blue and green, respectively. Caveolin-1 protein was located in intracellular membranes, as suggested by the inhomogeneous appearance of the immunolabeled membrane-embedded marker, and in the notochordal cell membrane.

The relative gene expression of *Caveolin-1 *in primary NCs on day 0 in culture was comparable to that in NC-rich NP tissue *ex vivo*, but thereafter increased significantly on days 2, 4, and 6 and remained stable on days 8 and 10 (see Additional files 7, 9 and 10). Caveolin-1 protein was located in intracellular membranes, as suggested by the inhomogeneous appearance of the immunolabeled membrane-embedded marker, and in the NC cell membrane (Figure 4). The expression of Caveolin-1 protein per cell was significantly higher on day 4 in culture than on days 0 and 2, but levels decreased thereafter on days 6, 8 and 10.

### The physiological role Caveolin-1 in the preservation of NCs was further investigated in Caveolin-1 KO mice

**Figure 5:**
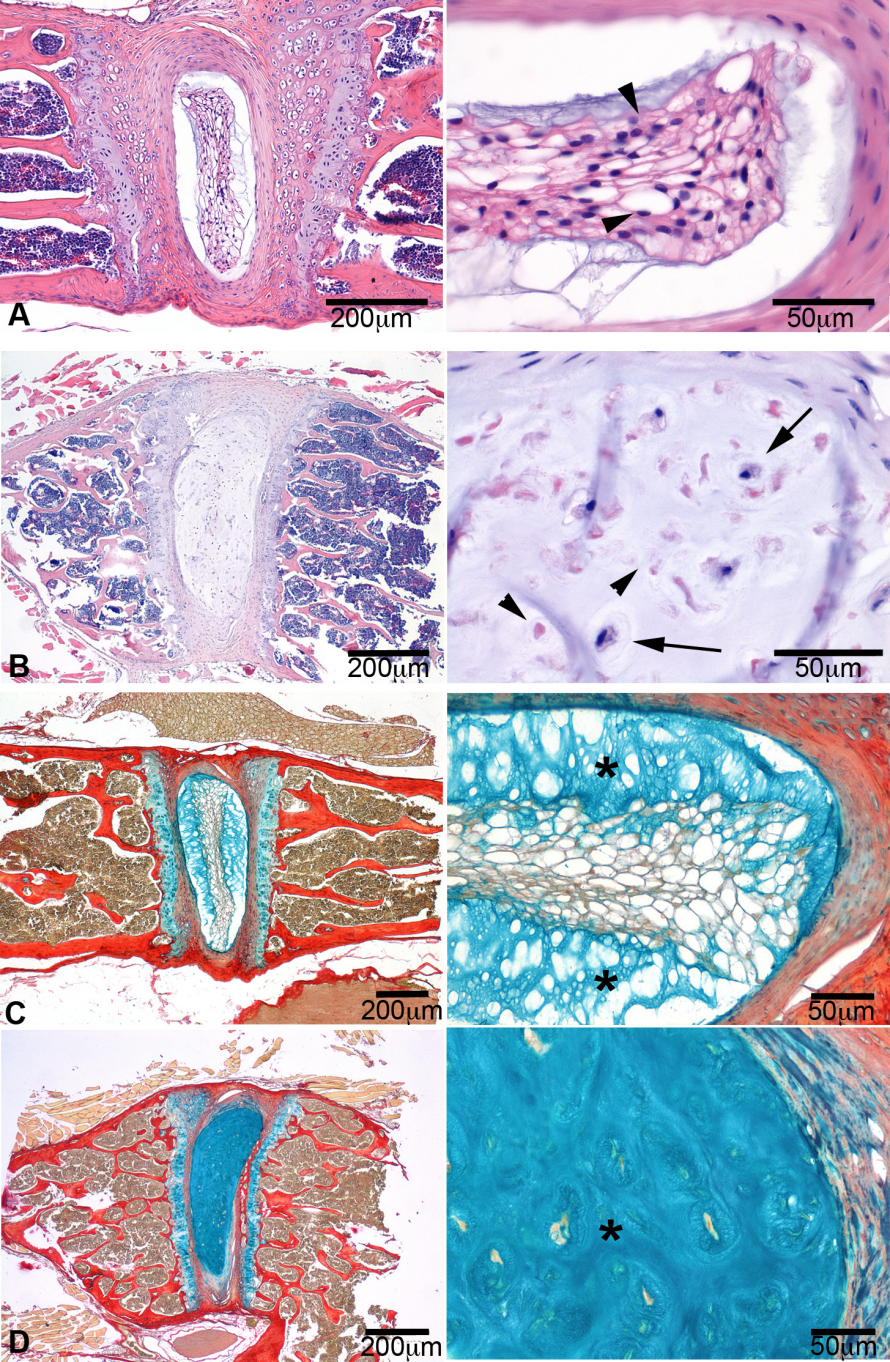
**The intervertebral disc phenotype of Caveolin-1 knock-out mice**. Typical examples of the intervertebral discs from 3-month-old wild-type (**A **and **C**) and Caveolin-1 knock-out (**B **and **D**) mice, stained with H&E (**A **and **B**) and alcian blue/pricrosirius red (**C **and **D**). The pictures on the right are magnifications of the corresponding pictures on the left. The wild-type nucleus pulposus (NP) consisted of centrally located, viable notochordal cells (arrowhead) and a relatively limited amount of chondroid-like intercellular matrix (*), which stains blue in the sections stained with alcian blue/picrosirius red. The NP of the Caveolin-1 KO mice contained apoptotic (arrowheads) and rounded cells, with a smaller amount of cytoplasm lacking the typical vacuolar aspect (arrows), and a relatively large amount of chondroid-like intercellular matrix (*).

The IVD of 3-month-old Caveolin-1 KO mice was significantly different from that of wild-type mice of the same age (Figure 5). The NP of the wild-type IVD consisted of a centrally located area of large cells with highly vacuolated cytoplasm and hyperchromatic nuclei, consistent with the morphological characteristics of viable NCs. A limited amount of chondroid-like intercellular matrix was visible within the area of NCs and there was a large rim of this matrix in the zone between the NCs and the endplate. In contrast, the NP of the Caveolin-1 KO mice contained rounded cells, with a smaller amount of cytoplasm lacking the typical vacuolar appearance. Over 75% of these cells did not contain recognizable nuclei and were characterized by cytoplasmic eosinophilia suggestive of necrosis or apoptosis. The NP of Caveolin-1 KO mice contained a large amount of chondroid-like intercellular matrix between the cells within the NP.

## Discussion

Gene expression profiling of chondroid metaplasia of the NP, which is seen in physiological aging as well in early (subclinical) degeneration of the IVD, revealed novel genes and pathways involved in the preservation of the NC in the healthy IVD. The results obtained indicate that IVD degeneration in dogs involved significant downregulation of Wnt/β-catenin signaling and Caveolin-1; the latter seems to be crucial for NC maintenance and an important factor in IVD degeneration.

Material was obtained from non-chondrodystrophic and chondrodystrophic dogs, a unique animal model in view of investigating the NC and degeneration/regeneration of the IVD: in non-chondrodystrophic dogs, the NC remains the predominant cell type of the NP during the majority of life, with IVD degeneration only occurring at old age and in selected IVDs; conversely, in chondrodystrophic dogs the NC is lost early in life, with concurrent degeneration of all IVDs. Therefore, these two dog types reflect a naturally occurring animal model representing differential maintenance of the NC and associated differences in maintenance of optimal matrix health of the IVD [14,30].

In the present study the main focus was the transition of a healthy NC-rich NP into an aging/degenerating CLC-rich NP. However, recent studies have indicated that both the healthy and degenerating NP, apart from NCs and CLCs, contain stem/progenitor cells (approximately 1% of the NP cell population) [75-77]. These NP progenitor cells express so-called stemness genes, and have been shown to functionally differentiate into the adipogenic, chondrogenic, and neurogenic lineage [75]. As these progenitor cells are present within the NP, it is very well possible that these cells had a profound effect upon the signaling of NCs and CLCs and on the studied biomolecular signaling events, particularly with respect to the different phenotypes of the non-chondrodystrophic and chondrodystrophic NP investigated. Additional studies are warranted on this specific matter.

The relative expression of the notochordal markers *Brachyury *and *Cytokeratin 8 *[12,37-39] revealed marked differences between chondrodystrophic and non-chondrodystrophic dogs. In the initial stages of early IVD degeneration (classified as the mixed group), *Brachyury *and *Cytokeratin 8 *gene expression remained constant, indicating the preservation of the NC phenotype in the NP at this stage in both chondrodystrophic and non-chondrodystrophic dogs. However, *Brachyury *and *Cytokeratin 8 *gene expression was significantly decreased in the CLC-rich NP from chondrodystrophic, but not non-chondrodystrophic dogs. Decreased expression of NC markers is also seen in degenerated human IVDs [78] and is indicative of a significant loss of NCs from the NP [12,37-39]. The maintenance of high levels of *Brachyury *and *Cytokeratin 8 *gene expression in the non-chondrodystrophic, CLC-rich NP indicates that the NP cells in non-chondrodystrophic dogs undergo significant morphological changes, but can preserve characteristics of NCs on the basis of their gene expression pattern. These findings are consistent with the observation that there are niches of NCs in degenerated, human CLC-rich NP [11,12,38,78]. Therefore, based on histopathological assessment and the expression of notochordal marker genes, and in accordance with previous studies [17,19,79,80], loss of the notochordal phenotype would appear to be associated with the accelerated IVD degeneration seen in chondrodystrophic dogs, whereas in non-chondrodystrophic dogs the NCs or CLCs retain notochordal characteristics, accounting for the relatively low prevalence of IVD degeneration observed in this breed type [22,81].

### Canonical Wnt signaling activity is decreased in early IVD degeneration, with a clear differences between non-chondrodystrophic and chondrodystrophic dogs

An increase in canonical Wnt signaling activity results in increased gene expression of *Axin2*, which is considered a reliable read-out for the activity of canonical Wnt signaling [69-71]. *Axin2 *gene expression was significantly downregulated in the CLC-rich NP compared with the NC-rich and mixed NP in both non-chondrodystrophic and chondrodystrophic dogs, indicating that early (subclinical) IVD degeneration involves a significant reduction in canonical Wnt signaling activity. These results seem contradictory to our previous findings [35], showing that the CLC-rich NP had a higher *Axin2 *gene expression than the NC-rich NP. However, in that particular study CLC-NP tissue from chondrodystrophic dogs was compared with NC-rich NP tissue from non-chondrodystrophic dogs. Indeed, chondrodystrophic NPs exhibit significantly higher levels of *Axin2 *gene expression than non-chondrodystrophic dogs in all histopathological groups investigated in this study (Figure 2), which explains the apparently contradictory results between the previous report [35] and the present one. The significantly higher *Axin2 *gene expression found in chondrodystrophic dogs was further sustained by the significantly higher signal intensity for β-catenin-protein expression within the CLC-rich NP tissue as shown by immunohistochemistry, and most probably less degraded β-catenin as shown by the western blot analysis (see Additional files 6 and 7).

The gene expression of *Wnt7b*, which activates canonical Wnt signaling through interacting with the Wnt receptors LRP5 and Fzd1 [72], was increased during NP chondrification and was consistently higher in all histopathological groups in non-chondrodystrophic dogs than in chondrodystrophic dogs. Increased *Wnt7b *gene expression has also been reported in the cartilage of patients with osteoarthritis (OA) and rheumatoid arthritis (RA) [82]. In that respect, the significantly higher gene expression of *Wnt7b *may reflect a response to increase canonical Wnt signaling activity and an attempt to preserve NP health. Interestingly, canonical Wnt signaling activity (*Axin2 *and *Fzd1 *expression) was markedly higher in chondrodystrophic dogs in all histopathological stages, as was also reported earlier in canine NP cells [35]. In chondrodystrophic dogs, the decrease in Wnt signaling activity during NP chondrification may be explained by the increased expression of *Wif1 *and *Frizzled-related protein (Frzb) *(Wnt inhibitors, Figure 2 see alos Additional file 3), and the reduced expression of *r-spondin-3 *(*rspo3; *Wnt activator, see Additional file 3). It is tempting to hypothesize about the potential role of Wnt signaling in the transition from the healthy, NC-rich NP to the aged or degenerated CLC-rich NP. As Wnt signaling regulates notochord fate and stem cell renewal and apoptosis [83,84], decreased Wnt signaling may result in increased apoptosis and decreased self-renewal of NCs or NP stem cells, and ultimately in chondroid metaplasia of the NP. The higher canonical Wnt signaling seen in chondrodystrophic dogs may reflect an ineffective attempt to preserve the notochordal phenotype of NP cells or to regulate stem cell renewal and apoptosis. Conversely, given the involvement of Wnt signaling in tissue degeneration [85,86], higher Wnt signaling in chondrodystrophic dogs might reflect a diminished capacity to limit Wnt signaling, resulting in accelerated extracellular matrix breakdown, as is observed in patients with OA or RA [82,85]. This line of thought may also be in accordance with recent findings showing increased β-catenin protein expression in degenerated human IVD tissue as compared with healthy controls [87]. Altogether, these data illustrate the dual role of Wnt signaling, which requires further elucidation with respect to the transition from the NC-rich to the CLC-rich NP, also with respect to the role played by progenitor cells within the NP in various stages of degeneration [75-77].

### Loss of NC phenotype involves significant downregulation of Caveolin-1 expression

Caveolin-1 is required for notochord development [88], and caveolins regulate canonical Wnt signaling by recruiting β-catenin to caveolae membranes, thereby inhibiting Wnt/β-catenin signaling and reinforcing cell-cell adhesion mechanisms [55], and by internalizing LRP6 (Wnt receptor), thereby activating canonical Wnt signaling [56]. Furthermore, Caveolin-1 stimulates canonical Wnt signaling by activating integrin-linked kinase which inhibits glycogen synthase kinase 3-β, a key enzyme in Wnt/β-catenin signaling that phosphorylates β-catenin leading to the subsequent degradation of this molecule [73]. Also, Caveolin-1 stimulates canonical Wnt signaling through the accumulation of β-catenin to caveolae membranes, thereby preventing degradation of β-catenin by glycogen synthase kinase 3-β [55]. The performed co-immunofluorescence analysis of Caveolin-1 and β-catenin (the effector protein of canonical Wnt signaling) showed protein expression peaks of both proteins localized at the cell membrane, which may indicate an interaction between these proteins within the NC. However, in CLCs no such protein expression/colocalization was observed. It is tempting to hypothesize that within the NC, caveolae function to regulate β-catenin signaling by preventing its degradation and to reinforce cell-cell clusters, which is a morphological characteristic of NCs [36]. In line with the decrease in canonical Wnt signaling in early IVD degeneration, *Caveolin-1 *gene expression was significantly downregulated in both breed types. Caveolin-1 protein expression was observed almost exclusively in NCs and decreased significantly with chondrification/degeneration of the NP (Figure 3). However, other authors have reported increased Caveolin-1 expression to be associated with senescence of NP cells [74,89] and chondrocytes, with subsequent IVD degeneration and OA [90], respectively. This discrepancy is probably because the other studies investigated advanced stages of degeneration involving CLCs only, whereas we investigated early degeneration, involving the transition from the NC-rich to CLC-rich NP. Apart from cell senescence, Caveolin is known to play a significant role in various cellular processes, including stem cell regulation and proliferation [91]. Therefore, Caveolin-1 may act differently according to the triggering signals and cellular context, such as the stage of IVD degeneration and in different cell types [92]. The relationship between Wnt/β-catenin signaling and Caveolin-1 in the NP in different stages of degeneration and in NCs compared with CLCs requires further investigation. Interestingly, the expression of Caveolin-1 protein was consistently lower in chondrodystrophic NP than in non-chondrodystrophic NP regardless of histopathological stage, indicating a direct relationship between the absence of Caveolin-1 and the accelerated loss of NCs from the NP in chondrodystrophic dogs.

### Caveolin-1 appears to fulfill essential functions in the NC cytoskeleton

In an attempt to understand the role of caveolin-1 in NC physiology, we studied its distribution in NCs in culture. Caveolin-1 protein was detected in the cell membrane and in intracellular membranes (Figure 4), as reported previously [93]. As expected, Caveolin-1 gene and protein expression increased significantly when the cells adhered to the culture plate (days 4 and 6). Caveolin interacts with actin filaments of the cytoskeleton [94] and its increased expression may be involved in the formation of the NC cytoskeleton and adherence of NCs to the culture plate. In accordance with these observations, microarray analysis revealed significant changes in cytoskeletal components, supporting a role for Caveolin-1 in the NC cytoskeleton. NCs are known to contain a dense network of intracellular Actin, which may be involved in the homeostasis of the intracellular vesicles and cluster formation of these cells [36]. An important function of Caveolin-1 may be to regulate the interaction of caveolae with the actin cytoskeleton, thereby controling whether caveolae are at the cell surface or traveling to interior sites, or regulating the homeostasis of intracellular vesicles and intercellular clusters [95].

### Absence of Caveolin-1 coincides with decreased NC preservation and early IVD degeneration

The essential role of Caveolin-1 in NC physiology was corroborated by the IVD phenotype of the Caveolin-1 KO mice. Unlike NP from wild-type mice, NP from Caveolin-1 KO mice showed relatively few healthy NC clusters; most NP cells lacked the morphological characteristics of NCs and showed signs of apoptosis, and the NP contained an abundance of intercellular chondroid matrix, similar to the CLC-rich NP (Figure 5). These changes are similar to the histopathological changes observed in the transition from an NC-rich to CLC-rich NP (Figure 1) [14,22]. Therefore, these findings suggest that Caveolin-1 is essential for NC maintenance, and that decreased Caveolin-1 expression is an important factor in NC physiology and IVD degeneration. To further investigate the role of Caveolin-1 and its relationship with Wnt/β-catenin, future studies need to focus on Wnt/β-catenin signaling in Caveolin-1 KO mice.

Apart from the involvement in IVD degeneration, Caveolin proteins are involved in stem cell regulation and proliferation, as well as in the pathogenesis of cancers, pulmonary hypertension, cardiomyopathy, diabetes, and muscular dystrophy [91]. There has been an increasing interest in the application of Caveolin-mimetic peptides for the treatment of both cancer and pulmonary hypertension [91]. The findings of this study suggest that Caveolin-1 is crucial for NC maintenance and IVD health, and this protein may be regarded an exciting target for developing ways to regenerate the IVD. For example, the degenerated IVD may be treated by locally applying Caveolin-1-mimetic peptide or by promoting the expression of Caveolin-1 in the cells of the degenerated IVD, thereby promoting regeneration of the degenerated tissue. Also, it should be taken into consideration that Caveolin-1 may exert different actions dependent on the cell context and the stage of degeneration [91], and further *in vitro *mechanistic studies are required to test this concept.

### Study limitations

In the present study the main focus was to investigate NCs and CLCs. However, the process of early IVD degeneration may also involve significant changes in NP progenitor/stem cells, and these cells may have a significant influence on the biomolecular signaling events within the NP.

A potential limitation of this study is that relatively few genes were differentially expressed when comparing the NC-rich, mixed, and CLC-rich NPs in the non-chondrodystrophic dogs, whereas in chondrodystrophic dogs relatively many genes were up- or downregulated. This might be because of the genetic heterogeneity of the non-chondrodystrophic sample (mongrels of different age and variable size), whereas the chondrodystrophic sample consisted of Beagle dogs of the same age and standardized size. For the chondrodystrophic breed group (Beagles), an appropriate sample size for the NC-rich, mixed, and CLC-rich groups could be obtained using young Beagles (25 to 36 months), as IVD degeneration occurs relatively early in life. In contrast, to obtain an appropriate sample size for the non-chondrodystrophic group, IVD material was obtained from young and older dogs, since most IVDs in these non-chondrodystrophic dogs remain rich in NCs and IVD degeneration mainly occurs at older age. Therefore, IVDs graded as mixed and CLC-rich could only be found in relatively older non-chondrodystrophic dogs, explaining the relatively large variation in age.

Apart from the distinct IVD phenotype, Caveolin-1 KO mice also exhibit a distinct bone phenotype [96]. Since the endplates are proposed to play a role in the pathophysiology of IVD degeneration, endplate changes may also influence IVD and NC physiology.

## Conclusions

Early (subclinical) degeneration of the IVD, which is characterized by changes in the NP cell population, involves significant changes in the expression of genes involved in canonical Wnt signaling, ultimately leading to downregulation of this pathway. The expression of Caveolin-1, which regulates canonical Wnt signaling, is decreased in the CLC-rich NP and appears to be essential to NC physiology and preservation. In view of the high resemblance between humans and dogs regarding the biochemistry and molecular biology of IVD degeneration, it is concluded that Caveolin-1 may play an important role in IVD aging/degeneration in humans as well. Caveolin-1 may serve as an interesting target for developing novel treatment strategies for IVD degeneration.

## Abbreviations

BSA: bovine serum albumin; Cav1: Caveolin-1; CLC: chondrocyte-like cell; DAB: diaminobenzidine; Dkk3: Dickkopf homolog 3; DMEM: Dubecco's modified Eagle's serum; EDTA: ethylenediaminetetraacetic acid; FCS: fetal calf serum; FDR: false discovery rate; Frzb: Frizzled related protein; FSUl: functional spine unit; GEO: Gene Expression Omnibus; Fzd1: Frizzled 1; H&E: hematoxylin and eosin; HRP: horseradish peroxidase; Ilk: integrin-linked kinase; IVD: intervertebral disc; KO: knock-out; Lrp5: Low density lipoprotein receptor-related protein 5; MAANOVA: microarray analysis of variance; MMP: matrix metalloproteinase; NC: notochordal cell; NP: nucleus pulposus; OA: osteoarthritis; PBS: phosphate-buffered saline; PLAU: plasminogen activator-urokinase; qPCR: quantitative polymerase chain reaction; RA: rheumatoid arthritis; RIPA: radioimmunoprecipitation assay; Rspo3: R-spondin-3; Wif1: Wnt inhibitory factor 1.

## Competing interests

The authors declare that there are no conflicts of interest to report.

## Authors' contributions

LS participated in the design and coordination of the study, carried out the material collection, RNA isolation, microarray results analysis, and multiphoton imaging, performed data analysis, and drafted the manuscript. BM participated in the design and coordination of the study, carried out the sample collection, and drafted the manuscript. DO performed the qPCR, immunohistochemistry and immunofluorescence experiments, and the associated data analyses. FR participated in the design of the study, performed the analysis of the microarray results, validated the qPCR experiments, and drafted the manuscript. NB participated in the design of the study, carried out the sample collection, and drafted the manuscript. RW designed the software program for analysis of immunofluorescence imaging, and drafted the manuscript. GG participated in the study design, performed all histopathological examinations, and drafted the manuscript. MH collected materials for the study, and drafted the manuscript. MG performed the microarray analyses and the associated statistics, and drafted the manuscript. DL performed the microarray analyses and the associated statistics, and drafted the manuscript. FH performed the microarray analyses and the associated statistics, and drafted the manuscript. HH participated in the design and coordination of the study, and drafted the manuscript. LC participated in the design and coordination of the study, and drafted the manuscript. LP participated in the design and coordination of the study, and drafted the manuscript. MT participated in the design and coordination of the study, carried out sample collection, performed analysis on the Caveolin KO mice and drafted the manuscript. All authors read and approved the manuscript.

## Supplementary Material

Additional file 1 **Table S1 Primers used for qPCR analysis**. Table describing the complete primer information for all target and reference genes investigated. Click here for file

Additional file 2 **Data analysis and statistics**. Complete description of the data analysis and statistics used for the microarray analyses and the qPCR, immunohistochemistry, and immunofluorescence analyses. Click here for file

Additional file 3 **Table S2 N-fold changes and *P*-values for microarray and qPCR analysis**. N-fold changes and corresponding *P*-values for micorarray and qPCR analysis of notochordal marker genes, caveolins, and genes involved in canonical Wnt signaling. Click here for file

Additional file 4 **Table S3 Top regulated genes for microarray analysis**. Results obtained for the microarray comparisons between the notochordal cell (NC)-rich nucleus pulposus (NP), mixed NP, and chondrocyte-like cell (CLC)-rich NP in non-chondrodystrophic and chondrodystrophic dogs, and between the breed types for each histological stage. Click here for file

Additional file 5 **Table S4 Top five most significantly regulated pathways**. Top five most significantly regulated pathways on the basis of all performed microarray comparisons using Metacore pathway analysis. Click here for file

Additional file 6 **Figure S1 Beta-catenin protein expression in the chondrocyte-like cell (CLC)-rich nucleus pulposus (NP) of non-chondrodystrophic and chondrodystrophic dogs**. Immunohistochemistry (typical examples and quantification of expression) and western blot analysis for β-catenin protein expression in the chondrocyte-like cell (CLC)-rich nucleus pulposus (NP). Click here for file

Additional file 7 **Figure legends for Additional files ****6****and ****9**. Figure legends for Additional file 6, Figure S1 and Additional file 9, Figure S2. Click here for file

Additional file 8 **Table S5 Linear mixed model results for Caveolin-1 immunohistochemistry**. Linear mixed model results for the Caveolin-1 immunohistochemistry analyses of healthy vs. early-degenerated nuclei pulposi in dogs with naturally occurring intervertebral disc degeneration. Click here for file

Additional file 9 **Figure S2 Caveolin-1 gene and protein expression in primary notochordal cells in monolayer culture**. Caveolin-1 gene and protein expression in primary notochordal cells in monolayer culture. Click here for file

Additional file 10 **Table S6 *P*-values for mixed model analaysis of Caveolin-1 expression in culture**. *P*-values for the mixed model analyses of Caveolin-1 gene and protein expression of notochordal cells in culture. Click here for file
