# Supplementary material for: Gene expression profiling of early intervertebral disc degeneration reveals a down-regulation of canonical Wnt signaling and caveolin-1 expression: implications for development of regenerative strategies
Source: Arthritis Res Ther. 2013 Jan 29;15(1):R23. doi: 10.1186/ar4157 (PMC3672710; doi:10.1186/ar4157)
Supplement: Additional file 1 — Table S1 Primers used for qPCR analysis. Table describing the complete primer information for all target and reference genes investigated. [file ar4157-S1.DOC]

**Additional file 1, Table S1. Primers used for qPCR analysis**

| **Gene name** | **Forward sequence 5’🡪3’** | | | **Exon** | **Reverse primer** | | | **Exon** | **Amplicon Size** | **Annealing Temp (^o^C)** | | **Accession no.** | |  | |
| --- | --- | --- | --- | --- | --- | --- | --- | --- | --- | --- | --- | --- | --- | --- | --- |
| ***Target Genes*** |  | | |  |  | | |  |  |  | |  | |  | |
| *t* | AGACAGCCAGCAATCTG | | | 5 | TGGAGGGAAGTGAGAGG | | | 6 | 115 | 53 | | NM_001003092.1 | |  | |
| *krt8* | CCTTAGGCGGGTCTCTCGTA | | | 9 | GGGAAGCTGGTGTCTGAGTC | | | 9 | 149 | 63 | | XM_543639.4 | |  | |
| *wnt3a* | TCCCTGCGTTTCCGAAG | | | 5 | GGACCGAACTTGGAGATGC | | | 5/6 | 89 | 64.5 | | XM_539327 | |  | |
| *wnt7b* | AACACGCACCAGTACACCAA | | | 4 | CACTTGCAGGTGAAGACCTC | | | 4 | 110 | 60 | | XM_539327 | |  | |
| *wif1* | CCGAAATGGAGGCTTTTGTA | | | 5 | ATGCAGAACCCAGGAGTGAC | | | 6 | 135 | 61.5 | | XM_538269.3 | |  | |
| *fzd1* | GGCGCAGGGCACCAAGAAG | | | 1 | GAGCGACAGAATCACCCACCAGA | | | 1 | 97 | 63 | | XM_539411 | |  | |
| *lrp5* | GATCCTTGTGTCCGATGACC | | | 8 | ACTCGATTTTGGGGTTTTCC | | | 9 | 104 | 53 | | XM_003432415 | |  | |
| *dkk3* | CATCCAGTCCAGTGCTCTCA | | | 3’ÚTR | GGGCCAGGATTGTAAGTGAA | | | 3’ÚTR | 140 | 58 | | XM_534060 | |  | |
| *ilk* | AAAGCAGGGACTTCAATGAGGA | | | 7 | ACTTCACAGCTTGGCTCTGG | | | 8 | 197 | 64 | | XM_858003 | |  | |
| *cav1* | CGCACACCAAGGAAATCG | | | 1 | AAATCAATCTTGACCACGTCG | | | 2 | 72 | 60 | | NM_001003296 | |  | |
| *axin2* | GGACAAATGCGTGGATACCT | | | 1 | TGCTTGGAGACAATGCTGTT | | | 1 | 141 | 60 | | XM_548025 | |  | |
| ***Reference Genes*** |  | | |  |  | | |  |  |  | |  | |  | |
| *hprt* | AGCTTGCTGGTGAAAAGGAC | | | 5/6 | TTATAGTCAAGGGCATATCC | | | 7 | 104 | 56 | | NM_001003357 | |  | |
| *rps19* | CCTTCCTCAAAAAGTCTGGG | | | 2/3 | GTTCTCATCGTAGGGAGCAAG | | | 3 | 95 | 61 | | XM_533657 | |  | |
| *srpr* | GCTTCAGGATCTGGACTGC | | | 7 | GTTCCCTTGGTAGCACTGG | | | 7/8 | 81 | 61 | | XM_546411 | |  | |
| *rpl13 [1]* | GCCGGAAGGTTGTAGTCGT | | | 3 | GGAGGAAGGCCAGGTAATTC | | | 4 | 87 | 61 | | XM_003432726 | |  | |
| *ywhaz [1]* | CGAAGTTGCTGCTGGTGA | | | 2 | TTGCATTTCCTTTTTGCTGA | | | 2/3 | 94 | 58 | | XM_533072 | |  | |
|  | |  |  | | |  |  | | | |  | |  |  |  |
|  | |  |  | | |  |  | | | |  | |  |  |  |

Primers (in-house design, except for [1]) used for qPCR analysis of *brachyury (t), cytokeratin 8 (krt8), wnt3a, wnt7b, wnt inhibitory factor 1 (wif1),* *frizzled 1 (fzd1), low density lipoprotein receptor-related protein 5 (lrp5), integrin-linked kinase (ilk), dickkopf homolog 3 (dkk3), caveolin-1 (cav1),* and *axin2*, and reference genes *hypoxanthine-guanine phosphoribosyltransferase* (*hprt*), *ribosomal protein S19* (*rps19*), *signal recognition particle receptor* (*srpr*), *ribosomal protein L13* (*rpl13*), and *tyrosine 3-monooxygenase/tryptophan 5-monooxygenase activation protein, zeta* (*ywhaz*). UTR = untranslated region.

1. Peters IR, Peeters D, Helps CR, Day MJ: **Development and application of multiple internal reference (housekeeper) gene assays for accurate normalisation of canine gene expression studies.** *Vet Immunol Immunopathol* 2007, **117:**55-66.
