# Supplementary material for: Gene expression profiling of early intervertebral disc degeneration reveals a down-regulation of canonical Wnt signaling and caveolin-1 expression: implications for development of regenerative strategies
Source: Arthritis Res Ther. 2013 Jan 29;15(1):R23. doi: 10.1186/ar4157 (PMC3672710; doi:10.1186/ar4157)
Supplement: Additional file 2 — Data analysis and statistics. Complete description of the data analysis and statistics used for the microarray analyses and the qPCR, immunohistochemistry, and immunofluorescence analyses. [file ar4157-S2.DOC]

**Additional file 2. Data analysis and statistics**

***Microarray analysis***: Patterns of gene expression were compared between the three stages of intervertebral disc (IVD) degeneration (notochordal cell-rich, mixed, chondrocyte-like cell-rich nucleus pulposus (NP)) within each breed type and between the two breed types. Data were analyzed using microarray analysis of variance (MAANOVA) [1]. In a fixed effect analysis, sample, array, and dye effects were modeled. P values were determined with a permutation F2-test, in which residuals were shuffled 5000 times globally. Genes with P < 0.05 after a Benjamini-Hochberg determination of false discovery rate (FDR) were considered significantly changed; a change cutoff value of 1.3-fold was applied. Resulting gene lists from the indirect comparisons between groups were converted to their human homologues and assigned to functional pathways using the GeneGo MetaCore platform [2].

***qPCR and immunohistochemistry/imunofluorescence****:* Statistical analyses were performed using R statistical software [3]. Linear mixed models [4], containing both fixed and random effects, were used to analyze the described parameters separately for the qPCR of tissue samples, immunohistochemistry, qPCR of culture samples, and immunofluorescence. The Akaike Information Criterion (AIC) was used for model selection. A random intercept for each dog was added to each model to take the correlation of the observations within a dog into account. If necessary, models were optimized by correcting for unequal variances and/or for autoregressive correlation. Conditions for the use of mixed models, including normal distribution of the data, were assessed by analyzing the residuals (PP- and QQ plots) of the acquired models; no violations of these conditions were observed. For all the below-described models, the Benjamini-Hochberg correction was used to correct for multiple comparisons [5]. *P*<0.05 was considered statistically significant.

In the qPCR analysis of tissue samples, the ΔΔCT for individual target genes was used as parameter value; relative gene expression data from all target genes were analyzed as one outcome variable because there were potential inter-gene correlations within the canonical Wnt-signaling pathway. The explanatory factors for the linear mixed model were ‘target gene’ (*brachyury, cytokeratin 8, wnt7b, wnt inhibitory factor 1* (*wif1*)*, frizzled 1* (*fzd1*)*, low density lipoprotein receptor-related protein 5* (*lrp5*)*,* *dickkopf homolog 3* (*dkk3*)*, integrin-linked kinase* (*ilk*)*, caveolin-1* (*cav1*) and (*axin2*), ‘degeneration stage’ (NC-rich, Mixed, CLC-rich), breed type (non-chondrodystrophic and chondrodystrophic), and the interaction between these factors. P values were calculated per target gene to analyze differences between groups and degeneration stages.

In the immunohistochemistry study, ‘integrated density’ and ‘mean gray value’ were calculated. The explanatory factors for the linear mixed model were ‘Group’ (NC-rich, Mixed, CLC-rich), ‘Breed’ (non-chondrodystrophic and chondrodystrophic), and the interaction between these factors. P values were calculated per parameter to analyze differences between groups and degeneration stages.

In the NC culture study, the parameter values used for qPCR and immunofluorescence of caveolin-1 were ΔCT for *caveolin-1* gene expression and caveolin-1 protein expression per cell, respectively. For the immunofluorescence analysis, the data obtained from 5 images for each time point per dog were used for data analysis. The fixed factor in the linear mixed models used for both parameters was ‘time point in culture’ (days 0, 2, 4, 6, 8 and 10). P values were calculated to analyze differences in caveolin-1 gene and protein expression between time points in culture.

**References**

1. Wu H, Kerr MK, Cui X, Churchill GA: **MAANOVA: a software package for the analysis of spotted cDNA microarray experiments.** In *The analysis of gene expression data: methods and software.* 2002

2. Ekins S, Nikolsky Y, Bugrim A, Kirillov E, Nikolskaya T: **Pathway mapping tools for analysis of high content data.** *Methods Mol Biol* 2007, **356:**319-350.

3. R Development Core Team: **R: A language and environment for statistical computing. R Foundation for Statistical Computing.** 2010.

4. Pinheiro J, Bates D, DebRoy S, D. S: **nlme: linear and nonlinear mixed effects models.** *R package version 3* 2009**:**1-96.

5. Benjamini Y, Hochberg Y: **Controlling the false discovery rate: a practical and powerful approach to multiple testing.** *J R Stat Soc B* 1995, **57:**289-300.
