# Supplementary material for: Gene expression profiling of early intervertebral disc degeneration reveals a down-regulation of canonical Wnt signaling and caveolin-1 expression: implications for development of regenerative strategies
Source: Arthritis Res Ther. 2013 Jan 29;15(1):R23. doi: 10.1186/ar4157 (PMC3672710; doi:10.1186/ar4157)
Supplement: Additional file 3 — Table S2 N-fold changes and P-values for microarray and qPCR analysis. N-fold changes and corresponding P-values for micorarray and qPCR analysis of notochordal marker genes, caveolins, and genes involved in canonical Wnt signaling. [file ar4157-S3.DOC]

| **Non-chondrodystrophic** | | | | | | | | | | | | | | | | | | | |  |
| --- | --- | --- | --- | --- | --- | --- | --- | --- | --- | --- | --- | --- | --- | --- | --- | --- | --- | --- | --- | --- |
|  | | **NC-rich vs. Mixed** | | | | | | **Mixed vs. CLC-rich** | | | | | | |  | **NC-rich vs. CLC-rich** | | | | |
|  | | **Array** | **P** | | **qPCR** | | **P** | |  | **Array** | **P** | | **qPCR** | **P** |  | **Array** | **P** | **qPCR** | **P** | |
| *t* | | -1.87 |  | | -1.02 | |  | |  | 1.31 |  | | 1.00 |  |  | -1.43 |  | -1.02 |  | |
| *krt8* | | -1.66 |  | | -1.27 | |  | |  | -1.95 |  | | 1.23 |  |  | -3.24 |  | -1.04 |  | |
| *wnt7b* | | -1.16 |  | | -1.21 | |  | |  | 1.10 |  | | **3.11** | **0.017** |  | -1.05 |  | 2.57 |  | |
| *wnt10b* | | 1.09 |  | |  | |  | |  | -1.08 |  | |  |  |  | 1.01 |  |  |  | |
| *wif1* | | 1.17 |  | | 2.20 | |  | |  | 1.81 |  | | -1.19 |  |  | 2.11 |  | 1.85 |  | |
| *rspo3* | | -2.41 |  | |  | |  | |  | 1.40 |  | |  |  |  | -1.73 |  |  |  | |
| *fzd1* | | -1.07 |  | | 1.48 | |  | |  | -1.00 |  | | -1.60 |  |  | -1.07 |  | -1.08 |  | |
| *frzb* | | 6.16 |  | |  | |  | |  | -1.17 |  | |  |  |  | 5.28 |  |  |  | |
| *lrp2* | | -1.38 |  | |  | |  | |  | 1.16 |  | |  |  |  | 1.16 |  |  |  | |
| *lrp5* | | -1.29 |  | | -1.73 | |  | |  | 1.24 |  | | 1.22 |  |  | -1.03 |  | -1.42 |  | |
| *ilk* | | -1.36 |  | | 1.33 | |  | |  | -0.81 |  | | -1.38 |  |  | -1.11 |  | -1.04 |  | |
| *dkk3* | | -1.03 |  | | 1.27 | |  | |  | -1.02 |  | | -1.82 |  |  | -1.05 |  | -1.44 |  | |
| *cav1* | | -1.37 |  | | -1.18 | |  | |  | -1.08 |  | | -1.01 |  |  | -1.47 |  | -1.20 |  | |
| *cav2* | | **-1.63** | **0.041** | |  | |  | |  | 1.46 |  | |  |  |  | -1.12 |  |  |  | |
| *cav3* | | 1.45 |  | |  | |  | |  | 1.26 |  | |  |  |  | 1.83 |  |  |  | |
| *axin2* | | 1.08 |  | | 2.58 | |  | |  | -1.01 |  | | **-2.95** | **0.025** |  | 1.07 |  | **-1.14** | **0.047** | |
|  | |  |  | |  | |  | |  |  |  | |  |  |  |  |  |  |  | |
|  | |  |  | |  | |  | |  | **Chondrodystrophic** | | | | |  |  |  |  |  | |
|  | | **NC-rich vs. Mixed** | | | | | | |  | **Mixed vs. CLC-rich** | | | | |  | **NC-rich vs. CLC-rich** | | | | |
|  | | **Array** | **P** | | **qPCR** | | **P** | |  | **Array** | | **P** | **qPCR** | **P** |  | **Array** | **P** | **qPCR** | **P** | |
| *t* | | **1.53** | **0.028** | | 1.67 | |  | |  | **-3.89** | | **0.001** | **-10.51** | **0.004** |  | **-2.54** | **0.017** | **-9.30** | **0.019** | |
| *krt8* | | -1.21 |  | | -1.16 | |  | |  | **-4.90** | | **0.031** | **-22.83** | **0.023** |  | **-5.94** | **0.026** | **-23.49** | **0.001** | |
| *wnt7b* | | **1.43** | **0.027** | | -3.13 | |  | |  | -1.19 | |  | **11.41** | **0.017** |  | 1.21 |  | 3.65 |  | |
| *wnt10b* | | **2.54** | **0.000** | |  | |  | |  | -1.23 | |  |  |  |  | **2.07** | **0.002** |  |  | |
| *wif1* | | -2.63 |  | | 1.57 | |  | |  | **3.64** | | **0.030** | **6.73** | **0.048** |  | 1.38 |  | **10.54** | **0.043** | |
| *rspo3* | | 1.48 |  | |  | |  | |  | **-5.14** | | **0.000** |  |  |  | **-3.48** | **0.001** |  |  | |
| *fzd1* | | 1.26 |  | | 1.49 | |  | |  | -1.08 | |  | 1.13 |  |  | 1.16 |  | 1.68 |  | |
| *frzb* | | -1.95 |  | |  | |  | |  | **14.18** | | **0.009** |  |  |  | **7.26** | **0.004** |  |  | |
| *lrp2* | | 1.42 |  | |  | |  | |  | **-2.58** | | **0.005** |  |  |  | -1.82 |  |  |  | |
| *lrp5* | | -1.11 |  | | 2.38 | |  | |  | **-2.46** | | **0.005** | -1.43 |  |  | **-2.74** | **0.001** | 1.66 |  | |
| *ilk* | | -1.11 |  | | 1.13 | |  | |  | **-1.45** | | **0.056** | 1.11 |  |  | **-1.61** | **0.049** | 1.25 |  | |
| *dkk3* | | 1.08 |  | | -1.05 | |  | |  | -1.07 | |  | 1.53 |  |  | 1.02 |  | 1.46 |  | |
| *cav1* | | -1.14 |  | | 1.12 | |  | |  | **-1.77** | | **0.017** | **-1.98** | **0.002** |  | **-2.02** | **0.011** | **-1.76** | **0.006** | |
| *cav2* | | 1.16 |  | |  | |  | |  | **-2.12** | | **0.009** |  |  |  | **-1.83** | **0.006** |  |  | |
| *cav3* | | **2.34** | **0.007** | |  | |  | |  | **1.67** | | **0.045** |  |  |  | **3.92** | **0.001** |  |  | |
| *axin2* | | 1.08 |  | | 1.13 | |  | |  | -1.10 | |  | **-1.77** | **0.025** |  | -1.02 |  | **-1.57** | **0.047** | |
|  | |  |  | |  | |  | |  |  | |  |  |  |  |  |  |  |  | |
| **Non-chondrodystrophic vs. chondrodystrophic** | | | | | | | | | | | | | | | | | | | | |
|  | **NC-rich** | | | | | | | |  | **Mixed** | | | | |  | **CLC-rich** | | | | |
|  | **Array** | | **P** | **qPCR** | | **P** | | |  | **Array** | | **P** | **qPCR** | **P** |  | **Array** | **P** | **qPCR** | **P** | |
| *t* | | -1.14 |  | 1.12 | | 0.983 | | |  | **2.52** | | **0.007** | 1.92 |  |  | -2.01 |  | **-8.09** | **0.024** | |
| *krt8* | | 1.05 |  | -1.30 | | 0.687 | | |  | 1.44 | |  | -1.18 |  |  | -1.75 |  | **-24.37** | **0.003** | |
| *wnt7b* | | -1.43 |  | **-2.94** | | **0.018** | | |  | 1.17 | |  | **-7.62** | **0.018** |  | -1.12 |  | **-2.08** | **0.018** | |
| *wnt10b* | | **-2.05** | **0.007** |  | |  | | |  | 1.13 | |  |  |  |  | -1.00 |  |  |  | |
| *wif1* | | 2.10 |  | 1.02 | | 0.584 | | |  | -1.47 | |  | -1.37 |  |  | 1.38 |  | **5.81** | **0.039** | |
| *rspo3* | | -1.44 |  |  | |  | | |  | 2.47 | |  |  |  |  | -2.90 |  |  |  | |
| *fzd1* | | -1.13 |  | **1.41** | | **0.042** | | |  | 1.20 | |  | **1.42** | **0.042** |  | 1.10 |  | **2.56** | **0.042** | |
| *frzb* | | 5.56 |  |  | |  | | |  | -2.16 | |  |  |  |  | 7.64 |  |  |  | |
| *lrp2* | | 1.51 |  |  | |  | | |  | **2.96** | | **0.004** |  |  |  | -1.01 |  |  |  | |
| *lrp5* | | 1.30 |  | -1.59 | | 0.458 | | |  | 1.50 | |  | 2.58 |  |  | -2.04 |  | 1.48 |  | |
| *ilk* | | 1.00 |  | 1.23 | | 0.051 | | |  | 1.23 | |  | 1.05 |  |  | -1.45 |  | 1.60 |  | |
| *dkk3* | | -1.15 |  | 1.19 | | 0.444 | | |  | -1.03 | |  | -1.12 |  |  | -1.08 |  | 2.49 |  | |
| *cav1* | | 1.02 |  | -1.21 | | 0.680 | | |  | 1.23 | |  | 1.09 |  |  | -1.34 |  | -1.79 |  | |
| *cav2* | | -1.01 |  |  | |  | | |  | **1.87** | | **0.046** |  |  |  | -1.65 |  |  |  | |
| *cav3* | | -1.93 |  |  | |  | | |  | -1.20 | |  |  |  |  | 1.11 |  |  |  | |
| *axin2* | | 1.29 |  | **5.40** | | **0.009** | | |  | 1.29 | |  | **2.36** | **0.009** |  | 1.18 |  | **3.92** | **0.009** | |

**Additional file 3, Table S2. N-fold changes and P values for microarray and qPCR analysis.**

N-fold changes and P values (in case of significance, highlighted in bold text) for microarray analysis of *brachyury (t), cytokeratin 8 (krt8),* and Wnt-associated gene targets *wnt7b, wnt10b, wnt inhibitory factor 1 (wif1), r-spondin-3* (*rspo3*), *frizzled 1 (fzd1), frizzled-related protein* (*frzb*), *low density lipoprotein receptor-related protein-2 (lrp2), low density lipoprotein receptor-related protein 5 (lrp5), integrin-linked kinase (ilk), dickkopf homolog 3 (dkk3), caveolin-1 (cav1), caveolin-2* (*cav2*), *caveolin-3* (*cav3*), and *axin2*, and qPCR of selected gene targets. N-fold changes are displayed for the comparisons between the Notochordal cell (NC)-rich nucleus pulposus (NP), the Mixed NP, and the chondrocyte-like cell (CLC)-rich NP, and between non-chondrodystrophic dogs and chondrodystrophic dogs for the three histological NP stages.
