# Supplementary material for: Gene expression profiling of early intervertebral disc degeneration reveals a down-regulation of canonical Wnt signaling and caveolin-1 expression: implications for development of regenerative strategies
Source: Arthritis Res Ther. 2013 Jan 29;15(1):R23. doi: 10.1186/ar4157 (PMC3672710; doi:10.1186/ar4157)
Supplement: Additional file 4 — Table S3 Top regulated genes for microarray analysis. Results obtained for the microarray comparisons between the notochordal cell (NC)-rich nucleus pulposus (NP), mixed NP, and chondrocyte-like cell (CLC)-rich NP in non-chondrodystrophic and chondrodystrophic dogs, and between the breed types for each histological stage. [file ar4157-S4.DOC]

| **Non-chondrodystrophic dogs: NC-rich NP (reference) vs. Mixed NP** | | | | | |
| --- | --- | --- | --- | --- | --- |
|  | | | | | |
| **Gene symbol** | **Description** | **GO term: Biological Process** | **Ensemble Gene ID** | **N-fold change** | **P-value** |
| CCL23 | Chemokine (C-C motif) ligand 23 | Cellular calcium ion homeostasis | ENSG00000167236 | 3.48 | 8.27E-03 |
| LYZ | Lysozyme | Cell wall macromolecule catabolic process | ENSG00000090382 | 3.41 | 8.92E-03 |
| BMP6 | Bone morphogenetic protein 6 | SMAD protein signal transduction | ENSG00000153162 | 3.26 | 2.98E-02 |
| SRGN | Serglycin | Negative regulation of bone mineralization | ENSG00000122862 | 2.59 | 2.98E-02 |
| PLBD1 | Phospholipase B domain containing 1 | Lipid catabolic process | ENSG00000121316 | 2.58 | 8.27E-03 |
| EMP2 | Epithelial membrane protein 2 | Cell proliferation | ENSG00000213853 | 2.40 | 3.65E-02 |
| EMB | Embigin | Cell adhesion | ENSG00000170571 | 2.34 | 2.88E-02 |
| VCAM1 | Vascular cell adhesion molecule 1 | Cell-cell adhesion | ENSG00000162692 | 2.27 | 8.53E-03 |
| CD53 | CD53 molecule | Signal transduction | ENSG00000143119 | 2.27 | 1.35E-02 |
| HPR | Haptoglobin-related protein | Photorespiration | ENSG00000257017 | 2.00 | 2.10E-02 |
| HP LOC100684963 | Hypothetical protein LOC100684963 | Not available | Not available | 1.94 | 2.10E-02 |
| PDE3B | Phosphodiesterase 3B, cGMP-inhibited | Glucose homeostasis | ENSG00000152270 | 1.89 | 2.10E-02 |
| STAT5B | Signal transducer and activator of transcription 5B | Regulation of steroid metabolic process | ENSG00000173757 | 1.63 | 4.07E-02 |
|  |  |  |  |  |  |
| CDH8 | Cadherin 8, type 2 | Cell-cell junction organization | ENSG00000150394 | -2.44 | 3.01E-03 |
| NUDT19 | Nudix (nucleoside diphosphate linked moiety X)-type motif 19 | Not available | ENSG00000213965 | -2.18 | 4.65E-02 |
| RINL | Ras and Rab interactor-like | Not available | ENSG00000187994 | -2.07 | 8.27E-03 |
| ST8SIA4 | ST8 alpha-N-acetyl-neuraminide alpha-2, 8-sialyltransferase 4 | Protein glycosylation | ENSG00000113532 | -1.97 | 2.10E-02 |
| LAMA4 | Laminin, alpha 4 | Regulation of cell adhesion | ENSG00000112769 | -1.94 | 8.53E-03 |
| RAB38 | RAB38, member RAS oncogene family | Small GTPase mediated signal transduction | ENSG00000123892 | -1.90 | 3.65E-02 |
| ITGA3 | Integrin, alpha 3 (antigen CD49C, alpha 3 subunit of VLA-3 receptor) | Cell adhesion | ENSG00000005884 | -1.81 | 2.10E-02 |
| MOSPD2 | Motile sperm domain containing 2 | Not available | ENSG00000130150 | -1.64 | 3.65E-02 |
| CAV2 | Caveolin 2 | Vesicle organization | ENSG00000105971 | -1.63 | 4.09E-02 |

**Additional file 4, Table S3. Top regulated genes for microarray analyses.**

| **Non-chondrodystrophic dogs: Mixed NP (reference) vs. CLC-rich NP** | | | | | |
| --- | --- | --- | --- | --- | --- |
| No significant regulations | | | | | |
|  |  |  |  |  |  |

| **Non-chondrodystrophic dogs: NC-rich NP (ref) vs. CLC-rich NP** | | | | | |
| --- | --- | --- | --- | --- | --- |
|  | | | | | |
| **Gene symbol** | **Description** | **GO: Biological Process** | **Ensemble Gene ID** | **N-fold change** | **P-value** |
| FAM63A | Family with sequence similarity 63, member A | Cellular Component | ENSG00000143409 | -2.88 | 3.46E-03 |

| **Chondrodystrophic dogs: NC-rich NP (reference) vs. Mixed NP** | | | | | | |
| --- | --- | --- | --- | --- | --- | --- |
| Total up-regulated genes: 570 | | | Total down-regulated genes: 712 | | | |
| **Gene Symbol** | **Description** | **GO term: Biological process** | | **Ensemble Gene ID** | **N-fold change** | **P-value** |
| SPSB2 | SplA/ryanodine receptor domain and SOCS box containing 2 | Intracellular signal transduction | | ENSG00000111671 | 7.28 | <1.00E-06 |
| ARHGEF10 | Rho guanine nucleotide exchange factor (GEF) 10 | Regulation of Rho protein signal transduction | | ENSG00000104728 | 4.88 | <1.00E-06 |
| TMEM93 | Transmembrane protein 93 | Not available | | ENSG00000127774 | 3.73 | <1.00E-06 |
| NDUFA7 | NADH dehydrogenase (ubiquinone) 1 alpha subcomplex, 7 | Oxidation-reduction process | | ENSG00000167774 | 3.57 | 7.11E-04 |
| SETD5 | SET domain containing 5 | Not available | | ENSG00000168137 | 3.41 | 4.02E-05 |
| FOLR4 | Folate receptor 4 (delta) homolog | Not available | | ENSG00000183560 | 3.27 | 3.75E-04 |
| ATM | Ataxia telangiectasia mutated | Reciprocal meiotic recombination | | ENSG00000149311 | 3.25 | 1.24E-02 |
| DERL2 | Der1-like domain family, member 2 | Response to unfolded protein | | ENSG00000072849 | 3.23 | 8.35E-04 |
| BICD1 | Bicaudal D homolog 1 | Anatomical structure morphogenesis | | ENSG00000151746 | 3.17 | 1.77E-04 |
| GOLGB1 | Golgin B1 | Golgi organization | | ENSG00000173230 | 3.06 | 3.21E-02 |
| SOCS1 | Suppressor of cytokine signaling 1 | Response to cytokine stimulus | | ENSG00000185338 | 2.98 | <1.00E-06 |
| EPHA4 | EPH receptor A4 | Cell adhesion | | ENSG00000116106 | 2.92 | 1.14E-05 |
| RBMX2 | RNA binding motif protein, X-linked 2 | Not available | | ENSG00000134597 | 2.78 | 2.02E-04 |
| C6orf118 | Chromosome 6 open reading frame 118 | Not available | | ENSG00000112539 | 2.72 | 3.05E-06 |
| FAM120A | Family with sequence similarity 120A | Not available | | ENSG00000048828 | 2.63 | 2.28E-04 |
| PCDH1 | Protocadherin 1 | Homophilic cell adhesion | | ENSG00000156453 | 2.60 | <1.00E-06 |
| C11orf91 | Chromosome 11 open reading frame 91 | Not available | | ENSG00000205177 | 2.55 | <1.00E-06 |
| WNT10B | Wingless-type MMTV integration site family, member 10B | Positive regulation of canonical Wnt receptor signaling pathway | | ENSG00000169884 | 2.54 | <1.00E-06 |
| ACADS | Acyl-CoA dehydrogenase, C-2 to C-3 short chain | Protein homotetramerization | | ENSG00000122971 | 2.46 | <1.00E-06 |
| BAZ1B | Bromodomain adjacent to zinc finger domain, 1B | Nucleosome disassembly | | ENSG00000009954 | 2.45 | 1.61E-04 |
| CCDC33 | Coiled-coil domain containing 33 | Not available | | ENSG00000140481 | 2.43 | 9.59E-04 |
| CFC1 | Cripto, FRL-1, cryptic family 1 | Determination of left/right symmetry | | ENSG00000136698 | 2.42 | <1.00E-06 |
| GORASP2 | Golgi reassembly stacking protein 2, 55kDa | Organelle organization | | ENSG00000115806 | 2.41 | <1.00E-06 |
| DST | Dystonin | Integrin-mediated signaling pathway | | ENSG00000151914 | 2.41 | <1.00E-06 |
| HMG20B | High mobility group 20B | Chromatin organization | | ENSG00000064961 | 2.40 | 1.06E-04 |
| C20orf43 | Chromosome 20 open reading frame 43 | Biological process | | ENSG00000022277 | 2.38 | 5.59E-05 |
| PGS1 | Phosphatidylglycerophosphate synthase 1 | Phospholipid biosynthetic process | | ENSG00000087157 | 2.36 | <1.00E-06 |
| FAM63B | Family with sequence similarity 63, member B | Biological process | | ENSG00000128923 | 2.35 | 2.64E-03 |
| CFL1 | Cofilin 1 (non-muscle) | Cytoskeleton organization | | ENSG00000172757 | 2.35 | 9.76E-04 |
| CAV3 | Caveolin 3 | Negative regulation of cardiac muscle hypertrophy | | ENSG00000182533 | 2.34 | 7.26E-03 |
| C6orf103 | Chromosome 6 open reading frame 103 | Proteolysis | | ENSG00000118492 | 2.33 | <1.00E-06 |
| TPCN2 | Two pore segment channel 2 | Trans membrane transport | | ENSG00000162341 | 2.31 | 8.65E-03 |
| ASTE1 | Asteroid homolog 1 (Drosophila) | DNA repair | | ENSG00000034533 | 2.30 | <1.00E-06 |
| MYO3B | Myosin IIIB | Response to stimulus | | ENSG00000071909 | 2.30 | 3.89E-05 |
| CSDE1 | Cold shock domain containing E1, RNA-binding | Regulation of transcription, DNA-dependent | | ENSG00000009307 | 2.29 | 5.16E-03 |
| NUAK2 | NUAK family, SNF1-like kinase, 2 | Actin cytoskeleton organization | | ENSG00000163545 | 2.26 | 1.21E-03 |
| SLC25A4 | Solute carrier family 25, member 4 | Transmembrane transport | | ENSG00000151729 | 2.22 | 1.05E-03 |
| TSPAN5 | Tetraspanin 5 | Not available | | ENSG00000168785 | 2.21 | 3.90E-02 |
| CCBL1 | Cysteine conjugate-beta lyase, cytoplasmic | Kynurenine metabolic process | | ENSG00000171097 | 2.19 | 9.91E-04 |
| DPEP3 | Dipeptidase 3 | Proteolysis | | ENSG00000141096 | 2.19 | 2.33E-04 |
| SLC7A1 | Solute carrier family 7, member 1 | Ion transport | | ENSG00000139514 | 2.18 | 6.58E-03 |
| ATG16L1 | ATG16 autophagy related 16-like 1 | Autophagic vacuole assembly | | ENSG00000085978 | 2.17 | 4.74E-05 |
| LAMA2 | Laminin, alpha 2 | Muscle organ development | | ENSG00000196569 | 2.17 | 5.87E-04 |
| LTF | Lactotransferrin | Ion transport | | ENSG00000012223 | 2.16 | 2.17E-03 |
| THRB | Thyroid hormone receptor, beta | Transcription, DNA-dependent | | ENSG00000151090 | 2.15 | 9.36E-03 |
|  |  |  | |  |  |  |
| YIPF2 | Yip1 domain family, member 2 | Not available | | ENSG00000130733 | -7.40 | <1.00E-04 |
| CCZ1 | CCZ1 vacuolar protein trafficking and biogenesis associated homolog | Vesicle docking | | ENSG00000122674 | -5.87 | <1.00E-04 |
| YPEL4 | Yippee-like 4 | Not available | | ENSG00000166793 | -4.83 | <1.00E-06 |
| ODAM | Odontogenic, ameloblast associated | Biomineral tissue development | | ENSG00000109205 | -4.64 | <1.00E-06 |
| SLC39A12 | Solute carrier family 39 (zinc transporter), member 12 | Zinc ion transport | | ENSG00000148482 | -4.57 | <1.00E-06 |
| TRIM45 | Tripartite motif containing 45 | Not available | | ENSG00000134253 | -4.48 | <1.00E-06 |
| VPS45 | Vacuolar protein sorting 45 homolog | Intracellular protein transport | | ENSG00000136631 | -4.10 | <1.00E-06 |
| SLC44A4 | Solute carrier family 44, member 4 | Transmembrane transport | | ENSG00000204385 | -4.05 | <1.00E-06 |
| KLHL9 | Kelch-like 9 (Drosophila) | Mitosis | | ENSG00000198642 | -3.98 | 2.78E-05 |
| WDFY1 | WD repeat and FYVE domain containing 1 | Biological process | | ENSG00000085449 | -3.89 | 1.61E-04 |
| RAB3A | RAB3A, member RAS oncogene family | Regulation of exocytosis | | ENSG00000105649 | -3.80 | <1.00E-06 |
| RTN4RL1 | Reticulon 4 receptor-like 1 | Axon regeneration | | ENSG00000185924 | -3.77 | <1.00E-06 |
| RTN1 | Reticulon 1 | Neuron differentiation | | ENSG00000139970 | -3.65 | <1.00E-06 |
| SYN3 | Synapsin III | Mitosis | | ENSG00000185666 | -3.42 | <1.00E-06 |
| MAT2A | Methionine adenosyltransferase II, alpha | Methylation | | ENSG00000168906 | -3.42 | <1.00E-06 |
| GPR35 | G protein-coupled receptor 35 | G-protein coupled receptor signaling pathway | | ENSG00000178623 | -3.34 | 1.10E-04 |
| MARCH4 | Membrane-associated ring finger (C3HC4) 4 | Biological Process | | ENSG00000144583 | -3.24 | <1.00E-06 |
| FGF13 | Fibroblast growth factor 13 | MAPKKK cascade | | ENSG00000129682 | -3.23 | <1.00E-06 |
| C5orf4 | Chromosome 5 open reading frame 4 | Fatty acid biosynthetic process | | ENSG00000170271 | -2.85 | <1.00E-06 |
| STK39 | Serine threonine kinase 39 | Protein phosphorylation | | ENSG00000198648 | -2.84 | <1.00E-06 |
| VCAN | Versican | Cell adhesion | | ENSG00000038427 | -2.73 | 1.58E-05 |
| MT2A | Metallothionein 2A | Cellular response to erythropoietin | | ENSG00000125148 | -2.70 | 8.65E-03 |
| SRRM3 | Serine/arginine repetitive matrix 3 | Not available | | ENSG00000177679 | -2.66 | <1.00E-06 |
| BICD2 | Bicaudal D homolog 2 (Drosophila) | microtubule anchoring at microtubule organizing center | | ENSG00000185963 | -2.65 | 5.43E-04 |
| IRF6 | Interferon regulatory factor 6 | Negative regulation of cell proliferation | | ENSG00000117595 | -2.65 | <1.00E-06 |
| IGSF5 | Immunoglobulin superfamily, member 5 | Cell-cell adhesion | | ENSG00000183067 | -2.63 | <1.00E-06 |
| CLEC3B | C-type lectin domain family 3, member B | Skeletal system development | | ENSG00000163815 | -2.61 | <1.00E-06 |
| MRPS10 | Mitochondrial ribosomal protein S10 | Translation | | ENSG00000048544 | -2.56 | 3.05E-06 |
| OR51V1 | Olfactory receptor, family 51, subfamily V, member 1 | Detection of chemical stimulus involved in sensory perception of smell | | ENSG00000176742 | -2.53 | 3.05E-06 |
| HDHD1 | Haloacid dehalogenase-like hydrolase domain containing 1 | Nucleotide metabolic process | | ENSG00000130021 | -2.53 | <1.00E-06 |
| ARFGAP2 | ADP-ribosylation factor GTPase activating protein 2 | Vesicle-mediated transport | | ENSG00000149182 | -2.52 | <1.00E-06 |
| OR10A7 | Olfactory receptor, family 10, subfamily A, member 7 | Response to stimulus | | ENSG00000179919 | -2.50 | 5.37E-03 |
| Cyp2c23 | Cytochrome P450, family 2, subfamily c, polypeptide 23 | Arachidonic acid metabolic process | | ENSRNOG00000013291 | -2.46 | <1.00E-06 |
| FAM3D | Family with sequence similarity 3, member D | Negative regulation of insulin secretion | | ENSG00000198643 | -2.45 | 5.98E-04 |
| H6PD | Hexose-6-phosphate dehydrogenase (glucose 1-dehydrogenase) | NADP metabolic process | | ENSG00000049239 | -2.41 | <1.00E-06 |
| SNRNP40 | Small nuclear ribonucleoprotein | MRNA processing | | ENSG00000060688 | -2.34 | <1.00E-06 |
| SGSM3 | Small G protein signaling modulator 3 | Cell cycle arrest | | ENSG00000100359 | -2.33 | <1.00E-06 |
| FLNB | Filamin B, beta | Cytoskeletal anchoring at plasma membrane | | ENSG00000136068 | -2.33 | <1.00E-06 |
| LSM3 | LSM3 homolog, U6 small nuclear RNA associated | mRNA processing | | ENSG00000170860 | -2.25 | <1.00E-06 |
| G2E3 | G2/M-phase specific E3 ubiquitin protein ligase | Protein polyubiquitination | | ENSG00000092140 | -2.23 | <1.00E-06 |
| APOA1BP | Apolipoprotein A-I binding protein | Biological process | | ENSG00000163382 | -2.23 | 3.28E-03 |
| TRIM38 | Tripartite motif containing 38 | Positive regulation of I-kappaB kinase/NF-kappaB cascade | | ENSG00000112343 | -2.22 | 3.59E-04 |
| EEF1A1 | Eukaryotic translation elongation factor 1 alpha 1 | Regulation of transcription, DNA-dependent | | ENSG00000156508 | -2.21 | 3.05E-06 |
| OR6C4 | Olfactory receptor, family 6, subfamily C, member 4 | Response to stimulus | | ENSG00000179626 | -2.19 | 6.38E-05 |
| TTC26 | Tetratricopeptide repeat domain 26 | Not available | | ENSG00000105948 | -2.19 | 3.05E-06 |

| **Chondrodystrophic dogs: Mixed NP (ref) vs. CLC-rich NP** | | | | | | |  |
| --- | --- | --- | --- | --- | --- | --- | --- |
| Total up-regulated genes: 1121 | | | Total down-regulated genes: 1173 | | | |  |
| **Gene Symbol** | **Description** | **GO term: Biological Process** | | **Ensemble Gene ID** | **N-fold change** | **P-value** |  |
| MT2A | Metallothionein 2A | Cellular response to erythropoietin | | ENSG00000125148 | 17.75 | 9.37E-04 |  |
| PRG4 | Proteoglycan 4 | Extracellular space | | ENSG00000116690 | 15.63 | 1.05E-03 |  |
| FRZB | Frizzled-related protein | Negative regulation of Wnt receptor signaling pathway | | ENSG00000162998 | 14.18 | 9.11E-03 |  |
| CPE | Carboxypeptidase E | Protein localization in membrane | | ENSG00000109472 | 11.97 | 2.80E-03 |  |
| CDO1 | Cysteine dioxygenase, type I | Sulfur amino acid biosynthetic process | | ENSG00000129596 | 11.14 | 2.35E-03 |  |
| DCN | Decorin | Peptide cross-linking via chondroitin 4-sulfate glycosaminoglycan | | ENSG00000011465 | 10.59 | 9.97E-03 |  |
| RBP4 | Retinol binding protein 4, plasma | Glucose homeostasis | | ENSG00000138207 | 9.73 | 6.66E-03 |  |
| LUM | Lumican | Collagen fibril organization | | ENSG00000139329 | 8.90 | 6.02E-03 |  |
| CP | Ceruloplasmin (ferroxidase) | Cellular iron ion homeostasis | | ENSG00000047457 | 8.89 | 9.81E-04 |  |
| NT5E | 5'-nucleotidase, ecto (CD73) | Nucleotide catabolic process | | ENSG00000135318 | 8.84 | 6.15E-04 |  |
| ADCY2 | Adenylate cyclase 2 (brain) | Activation of adenylate cyclase activity by G-protein signaling pathway | | ENSG00000078295 | 8.45 | 4.17E-03 |  |
| TSPAN13 | Tetraspanin 13 | Not available | | ENSG00000106537 | 8.11 | 1.35E-02 |  |
| NPNT | Nephronectin | Cell differentiation | | ENSG00000168743 | 7.88 | 2.68E-03 |  |
| RANBP3L | RAN binding protein 3-like | Intracellular transport | | ENSG00000164188 | 7.37 | 4.24E-02 |  |
| PID1 | Phosphotyrosine interaction domain containing 1 | Not available | | ENSG00000153823 | 7.32 | 1.65E-03 |  |
| CCDC152 | Coiled-coil domain containing 152 | Not available | | ENSG00000198865 | 7.01 | 3.02E-03 |  |
| PDPN | Podoplanin | Cell morphogenesis | | ENSG00000162493 | 6.91 | 8.32E-04 |  |
| SRGN | Serglycin | Negative regulation of bone mineralization | | ENSG00000122862 | 6.17 | 2.53E-02 |  |
| BBOX1 | Butyrobetaine (gamma), 2-oxoglutarate dioxygenase (gamma-butyrobetaine hydroxylase) 1 | Cellular nitrogen compound metabolic process | | ENSG00000129151 | 5.97 | 4.69E-03 |  |
| G25L | Glycoprotein 25L Precursor | Not available | | Not available | 5.95 | 9.19E-03 |  |
| SERPING1 | Serpin peptidase inhibitor, clade G (C1 inhibitor), member 1 | Negative regulation of endopeptidase activity | | ENSG00000149131 | 5.94 | 2.69E-03 |  |
| SPARCL1 | SPARC-like 1 (hevin) | Signal transduction | | ENSG00000152583 | 5.92 | 4.75E-03 |  |
| SMPDL3A | Sphingomyelin phosphodiesterase, acid-like 3A | Sphingomyelin catabolic process | | ENSG00000172594 | 5.80 | 4.74E-03 |  |
| COMP | Cartilage oligomeric matrix protein | Extracellular matrix | | ENSG00000105664 | 5.73 | 7.98E-04 |  |
| CXCR7 | Chemokine (C-X-C motif) receptor 7 | Negative regulation of apoptosis | | ENSG00000144476 | 5.66 | 2.25E-03 |  |
| SERPINA5 | Chemokineeptidase inhibitor, clade A (alpha-1 antiproteinase, antitrypsin), member 5 | Negative regulation of endopeptidase activity | | ENSG00000188488 | 5.65 | 3.24E-03 |  |
| SDC2 | Syndecan 2 | Response to hypoxia | | ENSG00000169439 | 5.65 | 3.90E-03 |  |
| MGP | Matrix Gla protein | Regulation of bone mineralization | | ENSG00000111341 | 5.60 | 9.44E-03 |  |
| ATP2B1 | ATPase, Ca++ transporting, plasma membrane 1 | ATP biosynthetic process | | ENSG00000070961 | 5.45 | 4.69E-03 |  |
| CENPP | Centromere protein P | Nucleosome assembly | | ENSG00000188312 | 5.40 | 1.63E-03 |  |
| PDK4 | Pyruvate dehydrogenase kinase, isozyme 4 | Regulation of acetyl-CoA biosynthetic process from pyruvate | | ENSG00000004799 | 5.21 | 3.38E-03 |  |
| MFAP5 | Microfibrillar associated protein 5 | Not available | | ENSG00000197614 | 5.19 | 1.24E-03 |  |
| BMP6 | Bone morphogenetic protein 6 | SMAD protein signal transduction | | ENSG00000153162 | 5.18 | 5.04E-03 |  |
| MN1 | Meningioma (disrupted in balanced translocation) 1 | Intramembranous ossification | | ENSG00000169184 | 5.17 | 2.45E-02 |  |
| SLC5A3 | Solute carrier family 5 (sodium/myo-inositol cotransporter), member 3 | Positive regulation of cell adhesion | | ENSG00000198743 | 5.15 | 1.18E-02 |  |
| SAA1 | Serum amyloid A1 | Biological process | | ENSG00000173432 | 4.99 | 6.82E-03 |  |
| TMEM50B | Transmembrane protein 50B | Polysaccharide metabolic process | | ENSG00000142188 | 4.96 | 9.95E-03 |  |
| CHST1 | Carbohydrate (keratan sulfate Gal-6) sulfotransferase 1 | Signal transduction | | ENSG00000175264 | 4.92 | 6.44E-04 |  |
| TNFRSF21 | Tumor necrosis factor receptor superfamily, member 21 | Positive regulation of cell migration | | ENSG00000146072 | 4.84 | 9.19E-03 |  |
| CXCL12 | Chemokine (C-X-C motif) ligand 12 | Collagen fibril organization | | ENSG00000107562 | 4.84 | 2.69E-03 |  |
| CPE | Carboxypeptidase E | Nitric oxide mediated signal transduction | | ENSG00000109472 | 4.77 | 3.38E-03 |  |
| COL2A1 | Collagen, type II, alpha 1 | Androgen receptor signaling pathway | | ENSG00000168542 | 4.77 | 4.02E-04 |  |
| Mt1 | Metallothionein-1-like | Prostaglandin biosynthetic process | | N/A | 4.73 | 6.32E-04 |  |
| PMEPA1 | Transmembrane prostate androgen-induced protein | Nervous system development | | ENSG00000124225 | 4.72 | 2.01E-03 |  |
| PTGES | Prostaglandin E synthase | Inflammation | | ENSG00000148344 | 4.71 | 5.35E-03 |  |
| SCRG1 | Stimulator of chondrogenesis 1 | Extracellular space | | ENSG00000164106 | 4.68 | 8.06E-03 |  |
|  |  |  | |  |  |  |  |
|  |  |  | |  |  |  |  |
| KRT18 | Keratin 18 | Golgi to plasma membrane CFTR protein transport | | ENSG00000111057 | -11.57 | 3.86E-03 |  |
| AKAP12 | A kinase (PRKA) anchor protein 12 | G-protein coupled receptor signaling pathway | | ENSG00000131016 | -8.07 | 1.90E-03 |  |
| MYL9 | Myosin, light chain 9, regulatory | Regulation of muscle contraction | | ENSG00000101335 | -7.21 | 2.00E-02 |  |
| PCDH15 | Protocadherin-related 15 | Cell adhesion | | ENSG00000150275 | -6.57 | 7.98E-04 |  |
| PLCL1 | Phospholipase C-like 1 | Lipid metabolic process | | ENSG00000115896 | -6.46 | 8.52E-03 |  |
| KRT19 | Keratin 19 | Sarcomere organization | | ENSG00000171388 | -5.79 | 3.90E-03 |  |
| APLN | Apelin | Positive regulation of phosphorylation | | ENSG00000143669 | -5.65 | 3.41E-03 |  |
| LYST | Lysosomal trafficking regulator | Endosome to lysosome transport via multivesicular body sorting pathway | | ENSG00000134762 | -5.62 | 3.08E-03 |  |
| MRPS27 | Mitochondrial ribosomal protein S27 | Not available | | ENSG00000113048 | -5.51 | 3.24E-03 |  |
| TSPAN7 | Tetraspanin 7 | Interspecies interaction between organisms | | ENSG00000156298 | -5.29 | 5.43E-03 |  |
| PKP2 | Plakophilin 2 | Carbohydrate metabolic process | | ENSG00000057294 | -5.26 | 5.65E-03 |  |
| TYW3 | tRNA-yW synthesizing protein 3 homolog (S. cerevisiae) | tRNA processing | | ENSG00000162623 | -5.24 | 2.90E-03 |  |
| NLGN4X | Neuroligin 4, X-linked | Cell-cell junction organization | | ENSG00000146938 | -5.21 | 4.02E-04 |  |
| RSPO3 | R-spondin 3 | Wnt receptor signaling pathway | | ENSG00000146374 | -5.14 | 2.03E-04 |  |
| ENPP2 | Ectonucleotide pyrophosphatase/phosphodiesterase 2 | Regulation of cell migration | | ENSG00000136960 | -5.14 | 8.09E-03 |  |
| SLC24A5 | Solute carrier family 24, member 5 | Ion transport | | ENSG00000188467 | -5.10 | 5.81E-03 |  |
| RAB38 | RAB38, member RAS oncogene family | Small GTPase mediated signal transduction | | ENSG00000123892 | -5.02 | 1.76E-03 |  |
| PCTP | Phosphatidylcholine transfer protein | Lipid transport | | ENSG00000141179 | -4.91 | 2.85E-03 |  |
| KRT8 | Keratin 8 | Cytoskeleton organization | | ENSG00000170421 | -4.90 | 3.08E-02 |  |
| KCNS3 | Potassium voltage-gated channel, delayed-rectifier, subfamily S, member 3 | Energy reserve metabolic process | | ENSG00000170745 | -4.75 | 7.06E-03 |  |
| SCRN1 | Secernin 1 | Exocytosis | | ENSG00000136193 | -4.73 | 2.13E-02 |  |
| THY1 | Thy-1 cell surface antigen | Cell-cell adhesion | | ENSG00000154096 | -4.72 | 4.91E-03 |  |
| LRRC4 | Leucine rich repeat containing 4 | Not available | | ENSG00000128594 | -4.69 | 2.80E-03 |  |
| XCL2 | Chemokine (C motif) ligand 2 | Chemotaxis | | ENSG00000143185 | -4.50 | 4.63E-02 |  |
| RAB20 | RAB20, member RAS oncogene family | Small GTPase mediated signal transduction | | ENSG00000139832 | -4.48 | 4.07E-03 |  |
| NAP1L1 | Nucleosome assembly protein 1-like 1 | Positive regulation of cell proliferation | | ENSG00000187109 | -4.41 | 1.76E-03 |  |
| SORBS2 | Sorbin and SH3 domain containing 2 | Biological process | | ENSG00000154556 | -4.39 | 1.19E-02 |  |
| NRG1 | Neuregulin 1 | Transcription, DNA-dependent | | ENSG00000157168 | -4.33 | 2.77E-03 |  |
| SPTLC3 | Serine palmitoyltransferase, long chain base subunit 3 | Sphingolipid metabolic process | | ENSG00000172296 | -4.26 | 1.63E-03 |  |
| PCDH20 | Protocadherin 20 | Cell adhesion | | ENSG00000197991 | -4.25 | 2.31E-03 |  |
| VWA5A | Von Willebrand factor A domain containing 5A | Not available | | ENSG00000110002 | -4.22 | 1.14E-03 |  |
| KIAA0368 | KIAA0368 | ER-associated protein catabolic process | | ENSG00000136813 | -4.12 | 4.13E-04 |  |
| CA8 | Carbonic anhydrase VIII | One-carbon metabolic process | | ENSG00000178538 | -4.12 | 2.63E-03 |  |
| CDH2 | Cadherin 2, type 1, N-cadherin (neuronal) | Regulation of Rho protein signal transduction | | ENSG00000170558 | -4.11 | 6.32E-04 |  |
| SORL1 | Sortilin-related receptor, L (DLR class) A repeats containing | Lipid transport | | ENSG00000137642 | -4.09 | 8.69E-03 |  |
| LAMB4 | Laminin, beta 4 | Cell adhesion | | ENSG00000091128 | -4.07 | 1.14E-03 |  |
| DSC2 | Desmocollin 2 | Cell adhesion | | ENSG00000134755 | -4.02 | 1.30E-02 |  |
| C10orf137 | Chromosome 10 open reading frame 137 | Regulation of transcription, DNA-dependent | | ENSG00000107938 | -3.98 | 1.39E-02 |  |
| CALD1 | Coiled-coil domain containing 152 | Actin filament bundle assembly | | ENSG00000122786 | -3.92 | 9.88E-03 |  |
| T | T, brachyury homolog (mouse) | Notochord development | | ENSG00000164458 | -3.89 | 1.32E-03 |  |
| SLC12A2 | Solute carrier family 12 (sodium/potassium/chloride transporters), member 2 | Ion transport | | ENSG00000064651 | -3.85 | 1.66E-02 |  |
| ADAMTS19 | ADAM metallopeptidase with thrombospondin type 1 motif, 19 | Proteolysis | | ENSG00000145808 | -3.75 | 4.57E-02 |  |
| CA2 | Carbonic anhydrase II | Carbon utilization | | ENSG00000104267 | -3.75 | 3.63E-02 |  |
| DSC3 | Desmocollin 3 | Cell adhesion | | ENSG00000134762 | -3.75 | 2.32E-03 |  |
| FAM184A | Family with sequence similarity 184, member A | Biological process | | ENSG00000111879 | -3.68 | 6.32E-04 |  |
| PCDH7 | Protocadherin-7 | Cell adhesion | | ENSG00000169851 | -3.68 | 7.22E-03 |  |
| FJX1 | Four-jointed box protein 1 | Not available | | ENSG00000179431 | -3.70 | 4.40E-03 |  |
| SPSB2 | SplA/ryanodine receptor domain and SOCS box containing 2 | Intracellular signal transduction | | ENSG00000111671 | -3.71 | 6.80E-03 |  |

| **Chondrodystrophic dogs: NC-rich NP vs. CLC-rich NP** | | | | | | |  |
| --- | --- | --- | --- | --- | --- | --- | --- |
| Total up-regulated genes: 1178 | | | Total down-regulated genes: 1741 | | | | |
| **Gene symbol** | **Description** | **GO term: Biological Process** | | **Ensemble Gene ID** | **N-fold Change** | **P-value** | |
| CPE | Carboxypeptidase E | Protein modification process | | ENSG00000109472 | 8.65 | 8.65E-05 | |
| TF | Transferrin | Transferrin transport | | ENSG00000091513 | 8.58 | 1.56E-02 | |
| CP | Ceruloplasmin (ferroxidase) | Cellular iron ion homeostasis | | ENSG00000047457 | 7.34 | 2.02E-03 | |
| FRZB | Frizzled-related protein | Negative regulation of canonical Wnt receptor signaling pathway | | ENSG00000162998 | 7.26 | 4.24E-03 | |
| DCN | Decorin | Peptide cross-linking via chondroitin 4-sulfate glycosaminoglycan | | ENSG00000011465 | 7.11 | 2.00E-02 | |
| COMP | Cartilage oligomeric matrix protein | Anti-apoptosis | | ENSG00000105664 | 7.08 | 6.69E-05 | |
| SRGN | Serglycin | Negative regulation of bone mineralization | | ENSG00000122862 | 6.97 | 9.64E-03 | |
| LUM | Lumican | Collagen fibril organization | | ENSG00000139329 | 6.88 | 1.39E-02 | |
| Not annotated | Not annotated | Not annotated | | Not annotated | 6.66 | 1.15E-02 | |
| MT2A | Metallothionein 2A | Cellular response to erythropoietin | | ENSG00000125148 | 6.60 | 5.33E-03 | |
| RANBP3L | RAN binding protein 3-like | Intracellular transport | | ENSG00000164188 | 6.54 | 4.71E-02 | |
| RBP4 | Retinol binding protein 4, plasma | Protein complex assembly | | ENSG00000138207 | 6.40 | 6.79E-04 | |
| CDO1 | Cysteine dioxygenase, type I | Response to glucagon stimulus | | ENSG00000129596 | 6.30 | 1.36E-02 | |
| ADCY2 | Adenylate cyclase 2 | Activation of adenylate cyclase activity by G-protein signaling pathway | | ENSG00000078295 | 5.97 | 3.94E-04 | |
| TSPAN13 | Tetraspanin 13 | Not available | | ENSG00000106537 | 5.88 | 1.95E-02 | |
| MFAP5 | Microfibrillar associated protein 5 | Not available | | ENSG00000197614 | 5.75 | 1.17E-04 | |
| PRG4 | Proteoglycan 4 | Cell proliferation | | ENSG00000116690 | 5.61 | 3.00E-03 | |
| S100A12 | S100 calcium binding protein A12 | Inflammatory response | | ENSG00000163221 | 5.59 | 1.15E-02 | |
| PID1 | Phosphotyrosine interaction domain containing 1 | Not available | | ENSG00000153823 | 5.57 | 8.41E-05 | |
| NPNT | Nephronectin | Cell differentiation | | ENSG00000168743 | 5.54 | 2.76E-04 | |
| LYZ | Lysozyme | Cell wall macromolecule catabolic process | | ENSG00000090382 | 5.47 | 2.42E-02 | |
| SPARCL1 | SPARC-like 1 (hevin) | Signal transduction | | ENSG00000152583 | 5.34 | 2.85E-02 | |
| G25L | Glycoprotein 25L | Not available | | Not available | 5.27 | 5.84E-04 | |
| SERPING1 | Serpin peptidase inhibitor, clade G (C1 inhibitor), member 1 | Regulation of proteolysis | | ENSG00000149131 | 5.08 | 6.92E-04 | |
| SMPDL3A | Sphingomyelin phosphodiesterase, acid-like 3A | Sphingomyelin catabolic process | | ENSG00000172594 | 5.01 | 1.16E-02 | |
| SERPINA5 | Serpin peptidase inhibitor, clade A (alpha-1 antiproteinase, antitrypsin), member 5 | Regulation of proteolysis | | ENSG00000188488 | 4.94 | 1.28E-03 | |
| BBOX1 | Butyrobetaine (gamma), 2-oxoglutarate dioxygenase (gamma-butyrobetaine hydroxylase) 1 | Cellular nitrogen compound metabolic process | | ENSG00000129151 | 4.83 | 2.82E-03 | |
| MT1 | Metallothionein 1 | Not available | | ENSG00000205360 | 4.82 | 6.04E-04 | |
| ARHGEF10 | Rho guanine nucleotide exchange factor (GEF) 10 | Regulation of Rho protein signal transduction | | ENSG00000104728 | 4.55 | < 1.0E-06 | |
| S100A9 | S100 calcium binding protein A9 | Chemotaxis | | ENSG00000163220 | 4.50 | 1.07E-02 | |
| PMEPA1 | Prostate transmembrane protein, androgen induced 1 | Androgen receptor signaling pathway | | ENSG00000124225 | 4.45 | 1.03E-04 | |
| SCRG1 | Stimulator of chondrogenesis 1 | Nervous system development | | ENSG00000164106 | 4.43 | 3.24E-03 | |
| SDC2 | Syndecan 2 | Response to hypoxia | | ENSG00000169439 | 4.34 | 5.78E-04 | |
| ATP2B1 | ATPase, Ca++ transporting, plasma membrane 1 | ATP biosynthetic process | | ENSG00000070961 | 4.21 | 1.31E-03 | |
| EPSTI1 | Epithelial stromal interaction 1 | Not available | | ENSG00000133106 | 4.21 | 1.62E-04 | |
| SPSB2 | SplA/ryanodine receptor domain and SOCS box containing 2 | Intracellular signal transduction | | ENSG00000111671 | 4.18 | < 1.0E-06 | |
| NT5E | 5'-nucleotidase, ecto (CD73) | Nucleotide catabolic process | | ENSG00000135318 | 4.14 | 4.17E-03 | |
| SMOC2 | SPARC related modular calcium binding 2 | Signal transduction | | ENSG00000112562 | 4.10 | 6.04E-04 | |
| CCL23 | Chemokine (C-C motif) ligand 23 | Cellular calcium ion homeostasis | | ENSG00000167236 | 4.09 | 1.67E-02 | |
| SLC5A3 | Solute carrier family 5 (sodium/myo-inositol cotransporter), member 3 | Transmembrane transport | | ENSG00000198743 | 3.96 | 1.66E-02 | |
| NDUFA7 | NADH dehydrogenase (ubiquinone) 1 alpha subcomplex | Oxidation-reduction process | | ENSG00000167774 | 3.93 | 3.42E-04 | |
| CAV3 | Caveolin 3 | Negative regulation of cardiac muscle hypertrophy | | ENSG00000182533 | 3.92 | 7.98E-04 | |
| PLBD1 | Phospholipase B domain containing 1 | Lipid catabolic process | | ENSG00000121316 | 3.87 | 1.20E-02 | |
| BCAT1 | Branched chain amino-acid transaminase 1, cytosolic | Branched chain family amino acid biosynthetic process | | ENSG00000060982 | 3.87 | 2.12E-02 | |
| S100A8 | S100 calcium binding protein A8 | Chemotaxis | | ENSG00000143546 | 3.85 | 8.49E-03 | |
| CAMP | Cathelicidin antimicrobial peptide | Defense response to bacterium | | ENSG00000164047 | 3.81 | 4.99E-02 | |
| CLIC6 | Chloride intracellular channel 6 | Ion transport | | ENSG00000159212 | 3.80 | 3.70E-04 | |
|  | | | | | | | |
| KRT18 | Keratin 18 | Golgi to plasma membrane CFTR protein transport | | ENSG00000111057 | -12.93 | 1.44E-03 | |
| TYW3 | tRNA-yW synthesizing protein 3 homolog | tRNA processing | | ENSG00000162623 | -7.83 | 9.45E-04 | |
| AKAP12 | A kinase (PRKA) anchor protein 12 | Signal transduction | | ENSG00000131016 | -7.69 | 7.56E-03 | |
| PLCL1 | Phospholipase C-like 1 | Lipid metabolic process | | ENSG00000115896 | -7.40 | 9.48E-03 | |
| DSC3 | Desmocollin 3 | Cell adhesion | | ENSG00000134762 | -7.05 | 1.40E-03 | |
| MYL9 | Myosin, light chain 9, regulatory | Regulation of muscle contraction | | ENSG00000101335 | -6.84 | 1.64E-02 | |
| MRPS27 | Mitochondrial ribosomal protein S27 | Not available | | ENSG00000113048 | -6.76 | 4.96E-04 | |
| ENPP2 | Ectonucleotide pyrophosphatase/phosphodiesterase 2 | Regulation of cell migration | | ENSG00000136960 | -6.41 | 9.38E-03 | |
| KRT19 | Keratin 19 | Cell differentiation involved in embryonic placenta development | | ENSG00000171345 | -6.32 | 6.00E-03 | |
| PKP2 | Plakophilin 2 | Carbohydrate metabolic process | | ENSG00000057294 | -6.13 | 6.02E-04 | |
| TSPAN7 | Tetraspanin 7 | Interspecies interaction between organisms | | ENSG00000156298 | -6.01 | 3.00E-03 | |
| KRT8 | Keratin 8 | Cytoskeleton organization | | ENSG00000170421 | -5.94 | 2.60E-02 | |
| NAP1L1 | Nucleosome assembly protein 1-like 1 | DNA replication | | ENSG00000187109 | -5.92 | 1.40E-03 | |
| RAB20 | RAB20, member RAS oncogene family | Small GTPase mediated signal transduction | | ENSG00000139832 | -5.79 | 2.91E-03 | |
| CALD1 | Caldesmon 1 | Positive regulation of protein binding | | ENSG00000122786 | -5.68 | 3.60E-03 | |
| KCNS3 | Potassium voltage-gated channel, delayed-rectifier, subfamily S, member 3 | Synaptic transmission | | ENSG00000170745 | -5.64 | 4.46E-03 | |
| APLN | Apelin | Positive regulation of phosphorylation | | ENSG00000171388 | -5.59 | 7.66E-03 | |
| SORBS2 | Sorbin and SH3 domain containing 2 | Biological process | | ENSG00000154556 | -5.42 | 3.39E-03 | |
| PCTP | Phosphatidylcholine transfer protein | Cholesterol metabolic process | | ENSG00000141179 | -5.37 | 1.30E-03 | |
| KCNIP1 | Kv channel interacting protein 1 | Synaptic transmission | | ENSG00000182132 | -5.14 | < 1.0E-06 | |
| CA2 | Carbonic anhydrase II | Carbon utilization | | ENSG00000104267 | -5.07 | 2.57E-02 | |
| THY1 | Thy-1 cell surface antigen | Cytoskeleton organization | | ENSG00000154096 | -4.74 | 1.10E-02 | |
| RAB38 | RAB38, member RAS oncogene family | GTP catabolic process | | ENSG00000123892 | -4.71 | 5.79E-04 | |
| SEMA3C | Sema domain, immunoglobulin domain (Ig), short basic domain, secreted, (semaphorin) 3C | Neural tube development | | ENSG00000075223 | -4.58 | 1.01E-04 | |
| DSC2 | Desmocollin 2 | Cell adhesion | | ENSG00000134755 | -4.48 | 9.91E-03 | |
| PHACTR1 | Phosphatase and actin regulator 1 | Regulation of transcription, DNA-dependent | | ENSG00000112137 | -4.48 | 3.17E-04 | |
| SCRN1 | Secernin 1 | Exocytosis | | ENSG00000136193 | -4.48 | 2.71E-02 | |
| SLC24A5 | Solute carrier family 24, member 5 | Ion transport | | ENSG00000188467 | -4.46 | 3.18E-03 | |
| CA3 | Carbonic anhydrase III, muscle specific | Response to oxidative stress | | ENSG00000164879 | -4.46 | 2.63E-03 | |
| TUBB2A | Tubulin, beta 2A | Microtubule-based movement | | ENSG00000137267 | -4.42 | 1.40E-02 | |
| BRAF | V-raf murine sarcoma viral oncogene homolog B1 | Negative regulation of apoptosis | | ENSG00000157764 | -4.39 | 8.65E-05 | |
| YIPF2 | Yip1 domain family, member 2 | Not available | | ENSG00000130733 | -4.35 | 3.12E-05 | |
| SLC12A2 | Solute carrier family 12 (sodium/potassium/chloride transporters), member 2 | Ion transport | | ENSG00000064651 | -4.34 | 1.10E-02 | |
| LGALS1 | Lectin, galactoside-binding, soluble, 1 | Positive regulation of I-kappaB kinase/NF-kappaB cascade | | ENSG00000100097 | -4.30 | 2.82E-03 | |
| C10orf137 | Chromosome 10 open reading frame 137 | Regulation of transcription, DNA-dependent | | ENSG00000107938 | -4.26 | 2.30E-02 | |
| LYST | Lysosomal trafficking regulator | Endosome to lysosome transport via multivesicular body sorting pathway | | ENSG00000143669 | -4.25 | 5.73E-03 | |
| ODAM | Odontogenic, ameloblast associated | Biomineral tissue development | | ENSG00000109205 | -4.25 | < 1.0E-06 | |
| PEX5L | Peroxisomal biogenesis factor 5-like | Protein import into peroxisome matrix | | ENSG00000114757 | -4.13 | 1.01E-04 | |
| PCDH7 | Protocadherin 7 | Cell adhesion | | ENSG00000169851 | -4.12 | 2.32E-03 | |
| EML2 | Echinoderm microtubule associated protein like 2 | Sensory perception | | ENSG00000125746 | -4.07 | 8.98E-03 | |
| PCDH15 | Protocadherin-related 15 | Cell adhesion | | ENSG00000150275 | -4.07 | 4.16E-03 | |
| KIAA0368 | KIAA0368 | ER-associated protein catabolic process | | ENSG00000136813 | -4.05 | 2.43E-05 | |
| VCAN | Versican | Cell adhesion | | ENSG00000038427 | -4.01 | < 1.0E-06 | |
| STX8 | Syntaxin 8 | Transport | | ENSG00000170310 | -3.99 | 8.47E-04 | |
| LRRC4 | Leucine rich repeat containing 4 | Not available | | ENSG00000128594 | -3.99 | 1.20E-03 | |
| C3orf49 | Chromosome 3 open reading frame 49 | Not available | | ENSG00000163632 | -3.98 | 6.41E-04 | |
| LAMB4 | Laminin, beta 4 | Cell adhesion | | ENSG00000091128 | -3.97 | 1.58E-04 | |
| Ehd3 | EH-domain containing 3 | Endocytic recycling | | ENSG00000013016 | -3.95 | 3.97E-02 | |

| **NC-rich NP: Non-chondrodystrophic (ref) vs. Chondrodystrophic dogs** | | | | | | |
| --- | --- | --- | --- | --- | --- | --- |
| Total up-regulated genes: 1241 | | | Total down-regulated genes: 562 | | | |
| **Gene symbol** | **Description** | **GO Biological Process** | | **Ensemble Gene ID** | **N-fold change** | **P-value** |
| BCL2 | B-cell CLL/lymphoma 2 | Apoptosis | | ENSG00000171791 | 3.03 | 1.28E-04 |
| BRI3BP | BRI3 binding protein | Not available | | ENSG00000184992 | 2.72 | 1.03E-04 |
| C5orf4 | Chromosome 5 open reading frame 4 | Fatty acid biosynthetic process | | ENSG00000170271 | 2.72 | 2.20E-02 |
| CALN1 | Calneuron 1 | Not available | | ENSG00000183166 | 2.61 | 1.50E-03 |
| CCK | Cholecystokinin | Positive regulation of cell proliferation | | ENSG00000187094 | 3.03 | 1.20E-03 |
| CCZ1 | CCZ1 vacuolar protein trafficking and biogenesis associated homolog (S. cerevisiae) | Vesicle docking | | ENSG00000122674 | 3.70 | 2.84E-02 |
| CILP | Cartilage intermediate layer protein, nucleotide pyrophosphohydrolase | Negative regulation of insulin-like growth factor receptor signaling pathway | | ENSG00000138615 | 4.37 | 3.12E-02 |
| COL3A1 | Collagen, type III, alpha 1 | Collagen fibril organization | | ENSG00000168542 | 2.68 | 2.38E-02 |
| CP | Ceruloplasmin (ferroxidase) | Ion transport | | ENSG00000047457 | 2.60 | 4.95E-02 |
| DENND4A | DENN/MADD domain containing 4A | Regulation of transcription, DNA-dependent | | ENSG00000174485 | 2.63 | <0.00E-04 |
| FGF13 | Fibroblast growth factor 13 | MAPKKK cascade | | ENSG00000129682 | 2.91 | 6.76E-03 |
| FLCN | Folliculin | Regulation of protein phosphorylation | | ENSG00000154803 | 3.10 | <0.00E-04 |
| FUT8 | Fucosyltransferase 8 (alpha (1,6) fucosyltransferase) | Protein glycosylation in Golgi | | ENSG00000033170 | 4.78 | 3.81E-04 |
| GNG2 | Guanine nucleotide binding protein (G protein), gamma 2 | GTP catabolic process | | ENSG00000186469 | 2.65 | 4.33E-04 |
| GREM1 | Gremlin 1 | Negative regulation of BMP signaling pathway | | ENSG00000166923 | 2.72 | <0.00E-04 |
| GRIK3 | Glutamate receptor, ionotropic, kainate 3 | Metabotropic glutamate receptor signaling pathway | | ENSG00000163873 | 2.69 | 1.99E-04 |
| GUCY1B3 | Guanylate cyclase 1, soluble, beta 3 | Nitric oxide mediated signal transduction | | ENSG00000061918 | 5.20 | <0.00E-04 |
| GULP1 | GULP, engulfment adaptor PTB domain containing 1 | Apoptosis | | ENSG00000144366 | 4.24 | <0.00E-04 |
| HSPH1 | Heat shock 105kDa/110kDa protein 1 | Chaperone mediated protein folding requiring cofactor | | ENSG00000120694 | 3.07 | <0.00E-04 |
| KLF4 | Kruppel-like factor 4 (gut) | Negative regulation of NF-kappaB transcription factor activity | | ENSG00000136826 | 2.65 | 1.17E-02 |
| KLHL9 | Kelch-like 9 (Drosophila) | Mitosis | | ENSG00000198642 | 3.57 | 2.77E-02 |
| MRPL23 | Mitochondrial ribosomal protein L23 | Mitochondrial translation | | ENSG00000214026 | 2.86 | 3.78E-03 |
| MSMO1 | Methylsterol monooxygenase 1 | Steroid metabolic process | | ENSG00000052802 | 3.46 | <0.00E-04 |
| MYLIP | Myosin regulatory light chain interacting protein | Positive regulation of protein catabolic process | | ENSG00000007944 | 2.69 | 6.61E-04 |
| NUDT4 | Nudix (nucleoside diphosphate linked moiety X)-type motif 4 | Cyclic nucleotide metabolic process | | ENSG00000173598 | 3.47 | 1.57E-04 |
| ODAM | Odontogenic, ameloblast asssociated | Biomineral tissue development | | ENSG00000109205 | 2.80 | 4.87E-02 |
| PDLIM3 | PDZ and LIM domain 3 | Actin filament organization | | ENSG00000154553 | 2.64 | 6.61E-04 |
| PDPN | Podoplanin | Cell morphogenesis | | ENSG00000162493 | 3.59 | 8.15E-03 |
| PNRC2 | Proline-rich nuclear receptor coactivator 2 | Deadenylation-independent decapping of nuclear-transcribed mRNA | | ENSG00000189266 | 2.68 | 1.05E-04 |
| PRPF38B | PRP38 pre-mRNA processing factor 38 (yeast) domain containing B | mRNA processing | | ENSG00000134186 | 2.81 | <0.00E-04 |
| RFTN2 | Raftlin family member 2 | Not available | | ENSG00000162944 | 4.28 | 5.50E-05 |
| RPL13A | Ribosomal protein L13a | Cytoplasmic translation | | ENSG00000142541 | 3.06 | 2.35E-02 |
| RTN1 | Reticulon 1 | Protein import into nucleus | | ENSG00000139970 | 2.86 | 9.50E-03 |
| SERPINI1 | Serpin peptidase inhibitor, clade I (neuroserpin), member 1 | Negative regulation of endopeptidase activity | | ENSG00000163536 | 3.62 | 6.68E-04 |
| SFMBT2 | Scm-like with four mbt domains 2 | Regulation of transcription, DNA-dependent | | ENSG00000198879 | 2.83 | 3.12E-04 |
| SLC38A2 | Solute carrier family 38, member 2 | Ion transport | | ENSG00000134294 | 3.45 | 1.05E-04 |
| SLC39A12 | Solute carrier family 39 (zinc transporter), member 12 | Ion transport | | ENSG00000148482 | 3.93 | 1.77E-03 |
| SLC44A4 | Solute carrier family 44, member 4 | Transmembrane transport | | ENSG00000204385 | 3.06 | 9.63E-03 |
| SPP1 | Secreted phosphoprotein 1 | Regulation of transcription, DNA-dependent | | ENSG00000118785 | 3.19 | 1.25E-02 |
| SQLE | Squalene epoxidase | Cholesterol biosynthetic proces | | ENSG00000104549 | 2.64 | 7.76E-05 |
| TRIM45 | Tripartite motif containing 45 | Not available | | ENSG00000134253 | 2.66 | 1.87E-02 |
| VCAN | Versican | Cell adhesion/Extracellular matrix | | ENSG00000038427 | 2.61 | 9.04E-03 |
| VEGFA | Vascular endothelial growth factor A | Cellular response to hypoxia | | ENSG00000112715 | 2.72 | 1.05E-04 |
| VPS45 | Vacuolar protein sorting 45 homolog (S. cerevisiae) | Golgi to vacuole transport | | ENSG00000136631 | 3.51 | 1.63E-03 |
| YIPF2 | Yip1 domain family, member 2 | Not available | | ENSG00000130733 | 4.07 | 1.23E-03 |
| YPEL4 | Yippee-like 4 (Drosophila) | Not available | | ENSG00000166793 | 3.09 | 1.10E-02 |
|  |  |  | |  |  |  |
| ARHGEF10 | Rho guanine nucleotide exchange factor (GEF) 10 | Regulation of Rho protein signal transduction | | ENSG00000104728 | -4.89 | 2.79E-03 |
| BICD1 | Bicaudal D homolog 1 | Minus-end-directed organelle transport along microtubule | | ENSG00000151746 | -2.38 | 4.00E-02 |
| C11orf91 | Chromosome 11 open reading frame 91 | Not available | | ENSG00000205177 | -2.25 | 1.12E-02 |
| CCDC33 | Coiled-coil domain containing 33 | Not available | | ENSG00000140481 | -2.72 | 2.82E-04 |
| CDH8 | Cadherin 8, type 2 | Cell-cell junction organization | | ENSG00000150394 | -2.25 | 1.35E-04 |
| CYFIP1 | Cytoplasmic FMR1 interacting protein 1 | Lamellipodium assembly | | ENSG00000068793 | -2.73 | 1.08E-02 |
| DERL2 | Der1-like domain family, member 2 | Response to unfolded protein | | ENSG00000072849 | -2.67 | 1.51E-02 |
| DPP10 | Dipeptidyl-peptidase 10 (non-functional) | Proteolysis | | ENSG00000175497 | -3.20 | 1.84E-03 |
| ENC1 | Ectodermal-neural cortex 1 (with BTB-like domain) | Multicellular organismal development | | ENSG00000171617 | -2.39 | 2.89E-02 |
| EPHA4 | EPH receptor A4 | Cell adhesion | | ENSG00000116106 | -2.26 | 4.76E-02 |
| ERRFI1 | ERBB receptor feedback inhibitor 1 | Regulation of Rho GTPase activity | | ENSG00000116285 | -3.21 | 3.66E-02 |
| FAM118A | Family with sequence similarity 118, member A | Not available | | ENSG00000100376 | -2.39 | 9.38E-04 |
| FHL2 | Four and a half LIM domains 2 | Negative regulation of transcription from RNA polymerase II promoter | | ENSG00000115641 | -2.81 | 2.86E-03 |
| FYTTD1 | Forty-two-three domain containing 1 | mRNA export from nucleus | | ENSG00000122068 | -2.59 | 1.59E-03 |
| GPN1 | GPN-loop GTPase 1 | Not available | | ENSG00000198522 | -3.29 | <0.00E-04 |
| GTPBP5 | GTP binding protein 5 (putative) | Ribosome biogenesis | | ENSG00000101181 | -2.42 | 2.07E-04 |
| H3F3B | H3 histone, family 3B | Nucleosome assembly | | ENSG00000132475 | -2.41 | 1.87E-02 |
| HIST1H2BA | Histone cluster 1, H2ba | Nucleosome assembly | | ENSG00000146047 | -3.94 | <0.00E-04 |
| HIST2H2BF | Histone cluster 2, H2bf | Nucleosome assembly | | ENSG00000203814 | -4.01 | <0.00E-04 |
| HS3ST1 | Heparan sulfate (glucosamine) 3-O-sulfotransferase 1 | Not available | | ENSG00000002587 | -2.29 | 3.79E-04 |
| Not available | Hypothetical protein LOC611312 | Not available | | Not available | -3.71 | 6.43E-05 |
| ITGB1BP2 | Integrin beta 1 binding protein (melusin) 2 | Signal transduction | | ENSG00000147166 | -3.02 | <0.00E-04 |
| JAG1 | Jagged 1 | Positive regulation of Notch signaling pathway | | ENSG00000101384 | -2.85 | 4.98E-04 |
| KCNJ10 | Potassium inwardly-rectifying channel, subfamily J, member 10 | Ion transport | | ENSG00000177807 | -2.31 | 5.07E-06 |
| LNX2 | Ligand of numb-protein X 2 | Protein homooligomerization | | ENSG00000139517 | -2.66 | <0.00E-04 |
| LRRC4C | Leucine rich repeat containing 4C | Regulation of axonogenesis | | ENSG00000148948 | -2.79 | 1.67E-03 |
| NDUFA7 | NADH dehydrogenase (ubiquinone) 1 alpha subcomplex | Oxidation-reduction proces | | ENSG00000167774 | -2.56 | 2.31E-02 |
| NFIA | Nuclear factor I/A | DNA replication | | ENSG00000162599 | -2.39 | 2.79E-03 |
| NFKBIA | Nuclear factor of kappa light polypeptide gene enhancer in B-cells inhibitor, alpha | Activation of NF-kappaB-inducing kinase activity | | ENSG00000100906 | -2.29 | 3.55E-02 |
| ODC1 | Ornithine decarboxylase 1 | Polyamine biosynthetic process | | ENSG00000115758 | -2.94 | 9.96E-06 |
| P4HB | Prolyl 4-hydroxylase, beta polypeptide | Lipid metabolic process | | ENSG00000185624 | -2.37 | 4.95E-02 |
| PGCP | Plasma glutamate carboxypeptidase | Tissue regeneration | | ENSG00000104324 | -2.29 | 5.65E-04 |
| PHLDA1 | Pleckstrin homology-like domain, family A, member 1 | Induction of apoptosis | | ENSG00000139289 | -2.76 | 2.01E-04 |
| PPAP2B | Phosphatidic acid phosphatase type 2B | Canonical Wnt receptor signaling pathway involved in positive regulation of cell-cell adhesion | | ENSG00000162407 | -4.55 | 1.82E-05 |
| PRC1 | Protein regulator of cytokinesis 1 | Vacuolar protein catabolic process | | ENSG00000198901 | -2.45 | 5.29E-04 |
| RBM11 | RNA binding motif protein 11 | Not avaiable | | ENSG00000185272 | -2.30 | 3.94E-02 |
| SETD5 | SET domain containing 5 | Not available | | ENSG00000168137 | -2.52 | 1.16E-02 |
| SOCS1 | Suppressor of cytokine signaling 1 | JAK-STAT cascade | | ENSG00000185338 | -2.65 | 2.38E-02 |
| SORBS1 | Sorbin and SH3 domain containing 1 | Positive regulation of glycogen biosynthetic process | | ENSG00000095637 | -3.15 | 5.07E-06 |
| SPSB2 | SplA/ryanodine receptor domain and SOCS box containing 2 | Intracellular signal transduction | | ENSG00000111671 | -6.24 | 1.10E-02 |
| TGFBR3 | Transforming growth factor, beta receptor III | Positive regulation of cell migration | | ENSG00000069702 | -2.77 | 3.14E-03 |
| TMEM93 | Transmembrane protein 93 | Not available | | ENSG00000127774 | -2.66 | 1.07E-02 |
| TNFRSF25 | Tumor necrosis factor receptor superfamily, member 25 | Cell surface receptor linked signaling pathway | | ENSG00000215788 | -2.32 | 1.20E-04 |
| TSPAN5 | Tetraspanin 5 | Not available | | ENSG00000168785 | -2.53 | 1.53E-02 |
| TUBB2A | Tubulin, beta 2A | Protein folding | | ENSG00000137267 | -4.39 | 6.43E-05 |

| **Mixed NP: Non-chondrodystrophic (ref) vs. Chondrodystrophic dogs** | | | | | | |
| --- | --- | --- | --- | --- | --- | --- |
| Total up-regulated genes: 604 | | | Total down-regulated genes: 248 | | | |
| **Gene Symbol** | **Description** | **GO Biological Process** | | **Ensemble Gene ID** | **N-fold change** | **P-value** |
| ATG7 | ATG7 autophagy related 7 homolog (S. cerevisiae) | Autophagy | | ENSG00000197548 | 2.72 | 6.15E-03 |
| ATL2 | Atlastin GTPase 2 | ER to Golgi vesicle-mediated transport | | ENSG00000119787 | 2.84 | < 1.00E-6 |
| ATP5A1 | ATP synthase, H+ transporting, mitochondrial F1 complex, alpha subunit 1, cardiac muscle | ATP metabolic process | | ENSG00000152234 | 3.00 | 0.00E+00 |
| CCL24 | Chemokine (C-C motif) ligand 24 | Positive regulation of cell migration | | ENSG00000106178 | 2.51 | 4.74E-04 |
| CSTF3 | Cleavage stimulation factor, 3' pre-RNA, subunit 3, 77kDa | mRNA processing | | ENSG00000176102 | 2.56 | 2.06E-04 |
| DSC2 | Desmocollin 2 | Cell adhesion | | ENSG00000134755 | 2.53 | 4.74E-04 |
| DSC3 | Desmocollin 3 | Protein stabilization | | ENSG00000134762 | 2.80 | 1.10E-02 |
| DSG1 | Desmoglein 1 | Cell-cell junction assembly | | ENSG00000134760 | 3.91 | 1.73E-04 |
| EDRF1 | Erythroid differentiation-related factor 1-like isoform 1 | Not available | | Not available | 2.30 | 2.96E-02 |
| EIF4G2 | Eukaryotic translation initiation factor 4 gamma, 2 | Regulation of translational initiation | | ENSG00000110321 | 2.54 | 5.90E-04 |
| FAM84B | Family with sequence similarity 84, member B | Not available | | ENSG00000168672 | 2.75 | 6.57E-03 |
| FBXO32 | F-box protein 32 | Protein ubiquitination | | ENSG00000156804 | 2.42 | 1.24E-04 |
| FLCN | Folliculin | Regulation of protein phosphorylation | | ENSG00000154803 | 2.54 | 3.56E-05 |
| FUT8 | Fucosyltransferase 8 (alpha (1,6) fucosyltransferase) | Protein glycosylation in Golgi | | ENSG00000033170 | 3.75 | 3.11E-05 |
| GNG2 | Guanine nucleotide binding protein (G protein), gamma 2 | GTP catabolic process | | ENSG00000186469 | 2.52 | 3.22E-03 |
| GNRH1 | Gonadotropin-releasing hormone 1 (luteinizing-releasing hormone) | Response to steroid hormone stimulus | | ENSG00000147437 | 2.55 | 1.73E-02 |
| GUCY1B3 | Guanylate cyclase 1, soluble, beta 3 | Nitric oxide mediated signal transduction | | ENSG00000061918 | 3.33 | 4.63E-02 |
| GULP1 | GULP, engulfment adaptor PTB domain containing 1 | Apoptosis | | ENSG00000144366 | 2.79 | < 1.00E-6 |
| HCFC2 | Host cell factor C2 | Regulation of transcription from RNA polymerase II promoter | | ENSG00000111727 | 2.40 | 2.97E-04 |
| HTRA1 | HtrA serine peptidase 1 | Negative regulation of BMP signaling pathway | | ENSG00000166033 | 2.46 | 2.18E-02 |
| KCNK2 | Potassium channel, subfamily K, member 2 | Potassium ion transmembrane transport | | ENSG00000082482 | 2.57 | 4.40E-03 |
| LAMB4 | Laminin, beta 4 | Cell adhesion | | ENSG00000091128 | 2.30 | 1.52E-02 |
| LDHB | Lactate dehydrogenase B | Glycolysis | | ENSG00000111716 | 2.50 | 4.74E-04 |
| LRP2 | Low density lipoprotein receptor-related protein 2 | Endocytosis | | ENSG00000081479 | 2.96 | 4.10E-03 |
| LYST | Lysosomal trafficking regulator | Endosome to lysosome transport via multivesicular body sorting pathway | | ENSG00000143669 | 3.44 | 2.57E-02 |
| MUS81 | MUS81 endonuclease homolog (S. cerevisiae) | Response to DNA damage stimulus | | ENSG00000172732 | 2.30 | 2.85E-04 |
| MYL6 | Myosin, light chain 6, alkali, smooth muscle and non-muscle | Muscle filament sliding | | ENSG00000196465 | 2.29 | < 1.00E-6 |
| NUDT4 | Nudix (nucleoside diphosphate linked moiety X)-type motif 4 | Cyclic nucleotide metabolic process | | ENSG00000173598 | 2.32 | 4.98E-05 |
| PCDH15 | Protocadherin-related 15 | Cell adhesion | | ENSG00000150275 | 3.24 | 1.45E-02 |
| PCDH20 | Protocadherin 20 | Cell adhesion | | ENSG00000197991 | 3.15 | 2.37E-02 |
| PDGFD | Platelet derived growth factor D | Regulation of peptidyl-tyrosine phosphorylation | | ENSG00000170962 | 2.34 | 1.66E-04 |
| PKP2 | Plakophilin 2 | Carbohydrate metabolic process | | ENSG00000057294 | 3.00 | 5.80E-03 |
| PLCL1 | Phospholipase C-like 1 | Lipid metabolic process | | ENSG00000115896 | 2.67 | 1.30E-02 |
| POPDC3 | Popeye domain containing 3 | Biological process | | ENSG00000132429 | 2.89 | 6.89E-04 |
| PTER | Phosphotriesterase related | Catabolic process | | ENSG00000165983 | 2.60 | 1.04E-02 |
| RAB27A | RAB27A, member RAS oncogene family | Protein transport | | ENSG00000069974 | 2.36 | 7.95E-04 |
| RAB38 | RAB38, member RAS oncogene family | Small GTPase mediated signal transduction | | ENSG00000123892 | 2.40 | 4.50E-02 |
| RSPO3 | R-spondin 3 | Wnt receptor signaling pathway | | ENSG00000146374 | 2.47 | 3.29E-02 |
| SPP1 | Secreted phosphoprotein 1 | Biomineral tissue development | | ENSG00000118785 | 2.81 | 4.80E-02 |
| SPTLC3 | Serine palmitoyltransferase, long chain base subunit 3 | Sphingolipid metabolic process | | ENSG00000172296 | 3.30 | 4.29E-03 |
| ST8SIA4 | ST8 alpha-N-acetyl-neuraminide alpha-2, 8-sialyltransferase 4 | Protein glycosylation | | ENSG00000113532 | 2.67 | 4.20E-04 |
| T | T, brachyury homolog (mouse) | Notochord development | | ENSG00000164458 | 2.52 | 7.47E-03 |
| TMEM45A | Transmembrane protein 45A | Not available | | ENSG00000181458 | 2.65 | 1.96E-03 |
| TRPM7 | Transient receptor potential cation channel, subfamily M, member 7 | Calcium-dependent cell-matrix adhesion | | ENSG00000092439 | 2.51 | 2.06E-04 |
| VWA5A | Von Willebrand factor A domain containing 5A | Not available | | ENSG00000110002 | 2.96 | 2.29E-02 |
| ZCCHC9 | Zinc finger, CCHC domain containing 9 | Negative regulation of phosphatase activity | | ENSG00000131732 | 2.48 | 1.66E-02 |
| ZNF323 | Zinc finger protein 323 | Viral reproduction | | ENSG00000235109 | 2.34 | < 1.00E-6 |
|  |  |  | |  |  |  |
|  |  |  | |  |  |  |
| MT2A | Metallothionein 2A | Cellular response to erythropoietin | | ENSG00000125148 | -7.81 | 2.93E-02 |
| AK5 | Adenylate kinase 5 | ADP biosynthetic process | | ENSG00000154027 | -2.11 | 3.60E-02 |
| AT1B1 | Sodium/potassium-transporting ATPase subunit beta-1 |  | | ENSG00000143153 | -2.04 | 3.24E-02 |
| ATP1A1 | ATPase, Na+/K+ transporting, alpha 1 polypeptide | ATP biosynthetic process | | ENSG00000163399 | -2.73 | 2.04E-02 |
| CD53 | CD53 molecule | Signal transduction | | ENSG00000143119 | -2.01 | 3.22E-03 |
| CKMT2 | Creatine kinase, mitochondrial 2 (sarcomeric) | Creatine metabolic process | | ENSG00000131730 | -2.72 | 1.56E-03 |
| COL4A1 | Collagen, type IV, alpha 1 | Cellular response to amino acid stimulus | | ENSG00000187498 | -2.92 | < 1.00E-6 |
| DES | Desmin | Cytoskeleton organization | | ENSG00000175084 | -4.00 | 2.21E-02 |
| DUSP6 | Dual specificity phosphatase 6 | Inactivation of MAPK activity | | ENSG00000139318 | -2.09 | 3.56E-05 |
| EMB | Embigin | Cell adhesion | | ENSG00000170571 | -2.08 | 1.25E-02 |
| ENC1 | Ectodermal-neural cortex 1 (with BTB-like domain) | Nervous system development | | ENSG00000171617 | -2.41 | 7.60E-03 |
| EPSTI1 | Epithelial stromal interaction 1 (breast) | Not available | | ENSG00000133106 | -2.81 | 4.57E-03 |
| GCKR | Glucokinase (hexokinase 4) regulator | Cellular glucose homeostasis | | ENSG00000084734 | -2.00 | 3.47E-02 |
| GDPD2 | Glycerophosphodiester phosphodiesterase domain containing 2 | Glycerol metabolic process | | ENSG00000130055 | -2.25 | 2.71E-02 |
| GLIPR1 | GLI pathogenesis-related 1 | Cellular lipid metabolic process | | ENSG00000139278 | -2.05 | 4.20E-04 |
| GNG11 | Guanine nucleotide binding protein (G protein), gamma 11 | GTP catabolic process | | ENSG00000127920 | -2.25 | 2.74E-02 |
| H3F3A | H3 histone, family 3A | Nucleosome assembly | | ENSG00000132475 | -2.03 | 2.85E-03 |
| HLA-DQB2 | Major histocompatibility complex, class II, DQ beta 2 | Immune response | | ENSG00000232629 | -2.08 | 2.41E-02 |
| HPR | Haptoglobin-related protein | Photorespiration | | ENSG00000257017 | -2.23 | 7.61E-04 |
| HP LOC100684119 | Hypothetical protein LOC100684119 | Not available | | Not available | -2.00 | 3.79E-02 |
| IL4 | Interleukin 4 | Positive regulation of T cell proliferation | | ENSG00000113520 | -2.05 | 3.80E-02 |
| KCNJ10 | Potassium inwardly-rectifying channel, subfamily J, member 10 | Potassium ion transport | | ENSG00000177807 | -2.15 | 1.22E-03 |
| LNX2 | Ligand of numb-protein X 2 | Protein homooligomerization | | ENSG00000139517 | -2.20 | 1.02E-02 |
| LRRC4C | Leucine rich repeat containing 4C | Regulation of axonogenesis | | ENSG00000148948 | -2.77 | 7.02E-03 |
| METTL7A | Methyltransferase like 7A | Metabolic process | | ENSG00000185432 | -2.11 | 2.08E-02 |
| MN1 | Meningioma (disrupted in balanced translocation) 1 | Intramembranous ossification | | ENSG00000169184 | -2.96 | 2.71E-02 |
| NFIL3 | Nuclear factor, interleukin 3 regulated | Transcription from RNA polymerase II promoter | | ENSG00000165030 | -2.57 | 1.10E-02 |
| NFKBIA | Nuclear factor of kappa light polypeptide gene enhancer in B-cells inhibitor, alpha | Activation of NF-kappaB-inducing kinase activity | | ENSG00000100906 | -2.38 | 9.21E-03 |
| PCK1 | Phosphoenolpyruvate carboxykinase 1 (soluble) | Oxaloacetate metabolic process | | ENSG00000124253 | -2.40 | 1.39E-03 |
| PDK4 | Pyruvate dehydrogenase kinase, isozyme 4 | Regulation of acetyl-CoA biosynthetic process from pyruvate | | ENSG00000004799 | -4.36 | 5.25E-04 |
| PLBD1 | Phospholipase B domain containing 1 | Lipid catabolic process | | ENSG00000121316 | -2.18 | 3.85E-02 |
| PLP1 | Proteolipid protein 1 | Protein integrin-mediated signaling pathway | | ENSG00000123560 | -2.15 | 3.63E-02 |
| PPAP2B | Phosphatidic acid phosphatase type 2B | Canonical Wnt receptor signaling pathway involved in positive regulation of cell-cell adhesion | | ENSG00000162407 | -2.78 | 7.09E-03 |
| PSPH | Phosphoserine phosphatase | Response to mechanical stimulus | | ENSG00000146733 | -2.39 | 5.37E-04 |
| PTPRO | Protein tyrosine phosphatase, receptor type, O | Peptidyl-tyrosine dephosphorylation | | ENSG00000151490 | -2.04 | 1.99E-02 |
| Q95JD6 | Sulfotransferase | Not available | | Not available | -2.32 | 2.10E-03 |
| RTN1 | Reticulon 1 | Protein import into nucleus | | ENSG00000139970 | -2.11 | 4.36E-02 |
| SLC30A1 | Solute carrier family 30 (zinc transporter), member 1 | Cadmium ion transmembrane transport | | ENSG00000170385 | -2.16 | 1.36E-03 |
| SRSF5 | Serine/arginine-rich splicing factor 5 | mRNA splice site selection | | ENSG00000100650 | -2.06 | 3.75E-03 |
| SVIL | Supervillin | Cytoskeleton organization | | ENSG00000197321 | -2.12 | 9.75E-04 |
| TGFBR3 | Transforming growth factor, beta receptor III | Epithelial to mesenchymal transition | | ENSG00000069702 | -2.73 | < 1.00E-6 |
| TXNDC11 | Thioredoxin domain containing 11 | Cell redox homeostasis | | ENSG00000153066 | -2.34 | 2.65E-04 |
| VCAM1 | Vascular cell adhesion molecule 1 | Cell-cell adhesion | | ENSG00000162692 | -2.14 | 2.62E-02 |
| ZBTB16 | Zinc finger and BTB domain containing 16 | Cartilage development | | ENSG00000109906 | -2.05 | 3.19E-02 |

| **CLC-rich NP: Non-chondrodystrophic (reference) vs. Chondrodystrophic dogs** | | | | | |
| --- | --- | --- | --- | --- | --- |
|  | | | | | |
| **Gene Symbol** | **Description** | **GO term: Biological Process** | **Ensemble Gene ID** | **N-fold change** | **P-value** |
| SERPINI1 | Serpin peptidase inhibitor, clade I (neuroserpin), member 1 | Regulation of cell adhesion | ENSG00000163536 | 5.18 | 3.08E-03 |
| C1orf63 | Chromosome 1 open reading frame 63 | Not available | ENSG00000117616 | 2.72 | 3.55E-03 |
| PRPF38B | PRP38 pre-mRNA processing factor 38 (yeast) domain containing B | mRNA processing | ENSG00000134186 | 2.30 | 1.36E-03 |
| C9orf156 | Chromosome 9 open reading frame 156 | Interspecies interaction between organisms | ENSG00000136932 | 2.21 | 3.55E-03 |
| TOB1 | Transducer of ERBB2, 1 | Negative regulation of BMP signaling pathway | ENSG00000141232 | 2.17 | 2.63E-03 |
| WDR67 | WD repeat domain 67 | Regulation of Rab GTPase activity | ENSG00000156787 | 1.98 | 3.55E-03 |
|  |  |  |  |  |  |
| HP LOC611312 | Hypothetical protein LOC611312 | Not available | Not available | -7.03 | 2.98E-03 |
| ERRFI1 | ERBB receptor feedback inhibitor 1 | Negative regulation of epidermal growth factor-activated receptor activity | ENSG00000116285 | -6.84 | 1.36E-03 |
| VEGFC | Vascular endothelial growth factor C | Vascular endothelial growth factor receptor signaling pathway | ENSG00000150630 | -3.25 | 2.98E-03 |
| PHYHD1 | Phytanoyl-CoA dioxygenase domain containing 1 | Not available | ENSG00000175287 | -2.16 | 2.98E-03 |

Results obtained for the microarray comparisons between the notochordal cell (NC)-rich nucleus pulposus (NP), Mixed NP, and chondrocyte-like cell (CLC)-rich NP in non-chondrodystrophic and chondrodystrophic dogs, and between the breed types for each histological stage. When >50 genes were regulated, the top 50 up- and

downregulated genes are displayed. For brevity, only one gene ontology (GO) term is displayed for each gene (obtained with bioDBnet [1]).

**References**

1. Mudunuri U, Che A, Yi M, Stephens RM: **bioDBnet: the biological database network.** *Bioinformatics* 2009, **25:**555-556.
