# Supplementary material for: Gene expression profiling of early intervertebral disc degeneration reveals a down-regulation of canonical Wnt signaling and caveolin-1 expression: implications for development of regenerative strategies
Source: Arthritis Res Ther. 2013 Jan 29;15(1):R23. doi: 10.1186/ar4157 (PMC3672710; doi:10.1186/ar4157)
Supplement: Additional file 5 — Table S4 Top five most significantly regulated pathways. Top five most significantly regulated pathways on the basis of all performed microarray comparisons using Metacore pathway analysis. [file ar4157-S5.DOC]

**Additional file 5, Table S4. Top 5 most significantly regulated pathways on the basis of all performed microarray comparisons using Metacore pathway analysis [1].**

| **Pathway** | **P value** |
| --- | --- |
| *Cytoskeleton remodeling: TGF,* ***Wnt*** *and cytoskeleton remodeling* | 2.63e-7 |
| Cell adhesion: extracellular matrix (ECM) remodeling | 8.22e-5 |
| Cell adhesion: plasmin signaling. | 1.58e-4 |
| Cell adhesion: plasminogen activator urokinase (PLAU) signaling. | 6.96e-4 |
| Bone Morphogenic Protein (BMP) signaling | 1.16e-2 |

**References**

1. Ekins S, Nikolsky Y, Bugrim A, Kirillov E, Nikolskaya T: **Pathway mapping tools for analysis of high content data.** *Methods Mol Biol* 2007, **356:**319-350.
