# Supplementary figures and images for: Gene expression profiling of early intervertebral disc degeneration reveals a down-regulation of canonical Wnt signaling and caveolin-1 expression: implications for development of regenerative strategies
Source: Arthritis Res Ther. 2013 Jan 29;15(1):R23. doi: 10.1186/ar4157 (PMC3672710; doi:10.1186/ar4157)

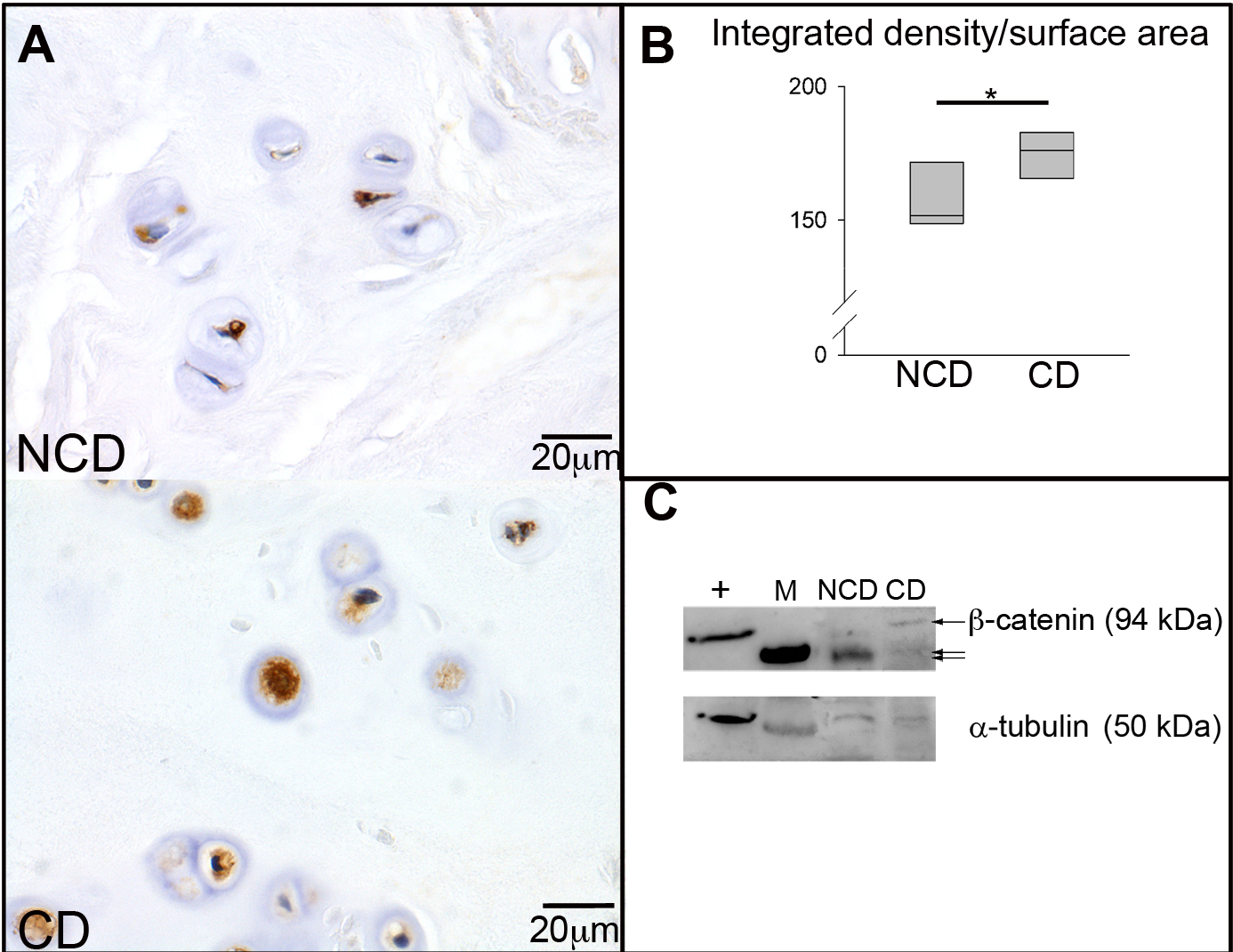

Supplement: Additional file 6 — Figure S1 Beta-catenin protein expression in the chondrocyte-like cell (CLC)-rich nucleus pulposus (NP) of non-chondrodystrophic and chondrodystrophic dogs. Immunohistochemistry (typical examples and quantification of expression) and western blot analysis for β-catenin protein expression in the chondrocyte-like cell (CLC)-rich nucleus pulposus (NP). [file ar4157-S6.TIFF]

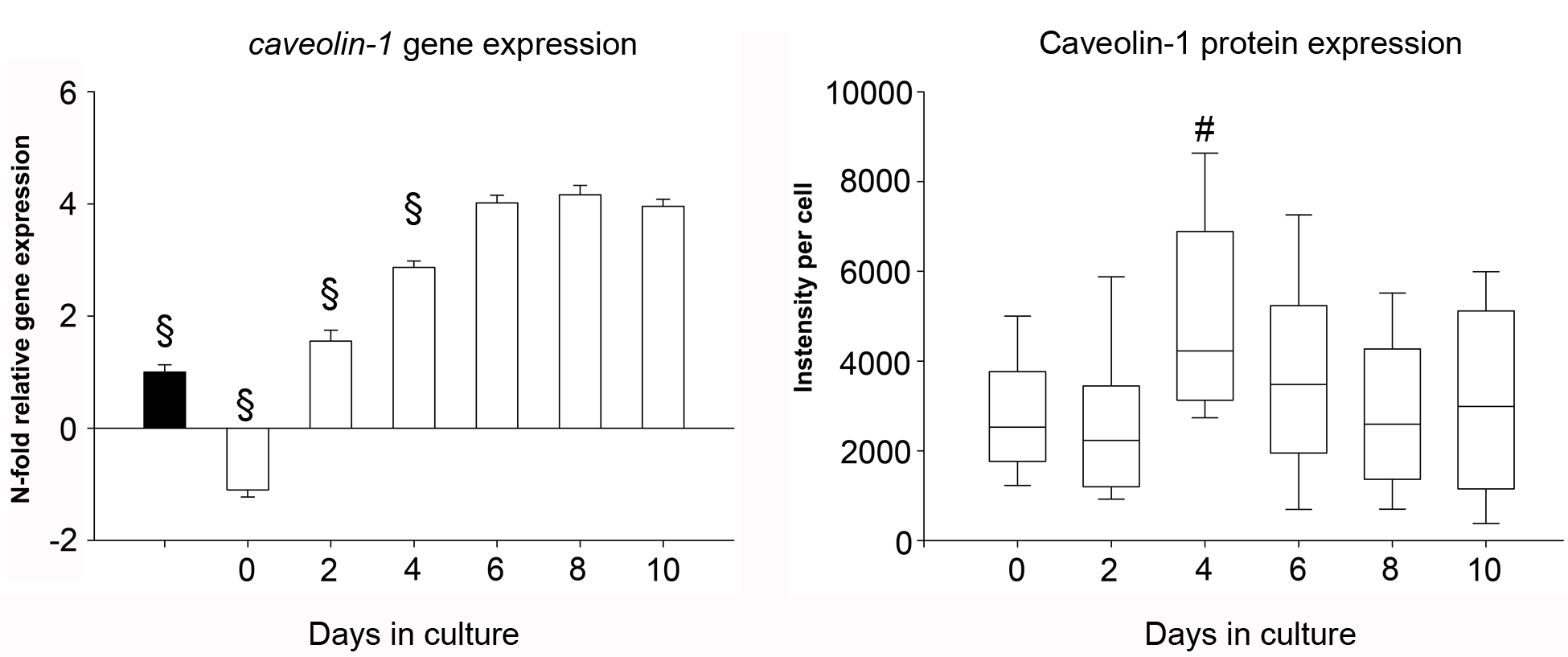

Supplement: Additional file 9 — Figure S2 Caveolin-1 gene and protein expression in primary notochordal cells in monolayer culture. Caveolin-1 gene and protein expression in primary notochordal cells in monolayer culture. [file ar4157-S9.TIFF]
