# Supplementary material for: Gene expression profiling of early intervertebral disc degeneration reveals a down-regulation of canonical Wnt signaling and caveolin-1 expression: implications for development of regenerative strategies
Source: Arthritis Res Ther. 2013 Jan 29;15(1):R23. doi: 10.1186/ar4157 (PMC3672710; doi:10.1186/ar4157)
Supplement: Additional file 7 — Figure legends for Additional files 6and 9. Figure legends for Additional file 6, Figure S1 and Additional file 9, Figure S2. [file ar4157-S7.DOC]

**Additional file 7: Figure legends for Additional files 6 and 9 (Figures S1 and S2)**

**Additional file 6, Figure S1. Beta-catenin protein expression in the chondrocyte-like cell (CLC)-rich nucleus pulposus (NP) of non-chondrodystrophic and chondrodystrophic dogs.** A) Typical examples of immunohistochemistry for β-catenin in the chondrocyte-like cell (CLC)-rich nucleus pulposus (NP) from non-chondrodystrophic (NCD) and chondrodystrophic (CD) dogs, showing a stronger staining intensity for β-catenin in CLCs of CD dogs. B) Boxplots for the integrated density/surface area of β-catenin staining for the CLC-rich NP of NCD and CD dogs. The asterisk indicates a significant difference between NCD and CD dogs. C) Western blot for β-catenin in the CLC-rich NP from NCD and CD dogs. Typically, in CD dogs β-catenin antibody identified the whole β-catenin (94 kDa; single arrow), whereas in NCD dogs a wider band of ~70 KDa (double arrow) was identified, most probably representing phosphorylated β-catenin subjected to degradation [1]. Protein extracted from the human insulinoma CM cell-line was used as positive control (+). Note that 5 μg protein of the CM cell-line was sufficient to generate a clear signal of β-catenin and α-tubulin, whereas 15 μg protein of NP extract gave a weak signal (also for α-tubulin) due to the relative abundance of matrix proteins within the CLC-rich NP.

1. Tesco G, Kim TW, Diehlmann A, Beyreuther K, Tanzi RE: **Abrogation of the presenilin 1/beta-catenin interaction and preservation of the heterodimeric presenilin 1 complex following caspase activation**. *J Biol Chem* 1998, **273**:33909-33914.

**Additional file 9, Figure S2. Caveolin-1 gene and protein expression in primary notochordal cells in monolayer culture.** A) Relative gene expression of *caveolin-1* in notochordal cell (NC) clusters on different days in culture. NC-rich NP tissue served as a reference (black bar, set at 1). §: indicates significant differences with all subsequent time points in culture. B) Boxplots of caveolin-1 protein expression intensity per cell on days 0, 2, 4, 6, 8, and 10 in NC clusters in culture. # indicates significant differences with all time points.
