# Supplementary material for: Gene expression profiling of early intervertebral disc degeneration reveals a down-regulation of canonical Wnt signaling and caveolin-1 expression: implications for development of regenerative strategies
Source: Arthritis Res Ther. 2013 Jan 29;15(1):R23. doi: 10.1186/ar4157 (PMC3672710; doi:10.1186/ar4157)
Supplement: Additional file 8 — Table S5 Linear mixed model results for Caveolin-1 immunohistochemistry. Linear mixed model results for the Caveolin-1 immunohistochemistry analyses of healthy vs. early-degenerated nuclei pulposi in dogs with naturally occurring intervertebral disc degeneration. [file ar4157-S8.DOC]

**Additional file 8, Table S5. Linear mixed model results for the caveolin-1 immunohistochemistry analyses of healthy vs. early-degenerated nuclei pulposi in dogs with naturally occurring intervertebral disc degeneration.**

| **Immunohistochemistry** | | | | | | | | |
| --- | --- | --- | --- | --- | --- | --- | --- | --- |
| *Parameter* | Group | | Breed | | | | Group*Breed | |
| Surface Area % | **0.006** | | 0.180 | | | | 0.063 | |
| Mean Grey Value | 0.967 | | **0.025** | | | | 0.750 | |
|  |  | | | |  | | |  |
| *Inter-group comparison per parameter* | | | | | | | | |
|  | | NCR-MX | | NCR-CLCR | | MX-CLCR | | |
| Surface Area % | | 0.052 | | **<0.001** | | **0.026** | | |
| Mean Grey Value | | - | | - | | - | | |

*P* values for the mixed model explanatory factors ‘Group’ (notochordal cell rich (NCR), mixed population (MX), chondrocyte-like cell rich (CLCR), ‘Breed’ (non-chondrodystrophic or chondrodystrophic dog), and their interactions. In case of significant main effects, *P* values for comparisons between groups per parameter and were calculated. *P*<0.05 was considered statistically significant.
