# Supplementary material for: Gene expression profiling of early intervertebral disc degeneration reveals a down-regulation of canonical Wnt signaling and caveolin-1 expression: implications for development of regenerative strategies
Source: Arthritis Res Ther. 2013 Jan 29;15(1):R23. doi: 10.1186/ar4157 (PMC3672710; doi:10.1186/ar4157)
Supplement: Additional file 10 — Table S6 P-values for mixed model analaysis of Caveolin-1 expression in culture. P-values for the mixed model analyses of Caveolin-1 gene and protein expression of notochordal cells in culture. [file ar4157-S10.DOC]

**Additional file 10, Table S6. *P* values for the mixed model analyses of caveolin-1 gene and protein expression of notochordal cells in culture**.

|  | ***caveolin-1***  **gene expression** | **caveolin-1**  **protein expression** |
| --- | --- | --- |
| NC tissue vs. Day 0 | 0.170 | - |
| NC tissue vs. Day 2 | **<0.001** | - |
| NC tissue vs. Day 4 | **<0.001** | - |
| NC Tissue vs. Day 6 | **<0.001** | - |
| NC tissue vs. Day 8 | **<0.001** | - |
| NC tissue vs. Day 10 | **<0.001** | - |
| Day 0 vs. 2 | **<0.001** | 0.630 |
| Day 0 vs. 4 | **<0.001** | **<0.001** |
| Day 0 vs. 6 | **<0.001** | 0.248 |
| Day 0 vs. 8 | **<0.001** | 0.519 |
| Day 0 vs. 10 | **<0.001** | 0.598 |
| Day 2 vs. 4 | **<0.001** | **<0.001** |
| Day 2 vs. 6 | **0.001** | 0.077 |
| Day 2 vs. 8 | **0.001** | 0.999 |
| Day 2 vs. 10 | **0.006** | 0.987 |
| Day 4 vs. 6 | **0.014** | **0.005** |
| Day 4 vs. 8 | **0.020** | **<0.001** |
| Day 4 vs. 10 | 0.124 | **<0.001** |
| Day 6 vs. 8 | 0.534 | 0.079 |
| Day 6 vs. 10 | 0.279 | 0.069 |
| Day 8 vs. 10 | 0.218 | 0.958 |

The explanatory variable used in the mixed model was ‘Time point’ (days 0, 2, 4, 6, 8 and 10 in culture), and *P* values for comparisons between time points were calculated. *P*<0.05 was considered statistically significant (highlighted in bold text).
